# *Anthonomus rubi* on Strawberry Fruit: Its Biology, Ecology, Damage, and Control from an IPM Perspective

**DOI:** 10.3390/insects12080701

**Published:** 2021-08-05

**Authors:** Lorenzo Tonina, Giulia Zanettin, Paolo Miorelli, Simone Puppato, Andrew G. S. Cuthbertson, Alberto Grassi

**Affiliations:** 1 Independent Agricultural Entomologists, Via Cavour, 22, 35020 Legnaro, PD, Italy; giuliazanettin22@gmail.com; 2 Fondazione Edmund Mach, 38098 San Michele all’Adige, TN, Italy; paolo.miorelli@fmach.it (P.M.); simone.puppato@fmach.it (S.P.); alberto.grassi@fmach.it (A.G.); 3 Independent Science Advisor, York YO10 5AQ, UK; andrew_cuthbertson@live.co.uk

**Keywords:** strawberry blossom weevil, host plant, sticky trap, mass trapping, bud removal, insecticide, groundcover, netting, adhesive tape, *Taraxacum* sp.

## Abstract

**Simple Summary:**

New damage on fruit caused by Strawberry Blossom Weevil (SBW) adults has been found in recent years in strawberry fields (soilless system under tunnel) in Trento Province, north-east Italy. According to this new scenario, studies on the biology, ecology, monitoring tools, and potential control methods for SBW were conducted to develop Integrated Pest Management (IPM) strategies. We observed the presence of SBW adults in strawberry fields all year round. In April, the young transplants are promptly visited by SBW adults. The first strawberry severed buds appear immediately after the development of the first flower trusses. Then from May until late October SBW damages the fruit. The mass trapping technique, using the green bucket traps baited with synthetic attractant, showed unsatisfactory results. In contrast, the same attractant combined with yellow or green sticky traps showed good efficacy in capturing adults. The high temperatures provided by the black fabric, the periodic removal of severed buds or adults and the Chlorpyrifos-methyl application constrained pest population build-up effectively. Our observations provide clarification of the new additional feeding habits of SBW and are fundamental in developing IPM strategies.

**Abstract:**

The strawberry blossom weevil (SBW), *Anthonomus rubi*, is a well-documented pest of strawberry. Recently, in strawberry fields of Trento Province (north-east Italy), new noteworthy damage on fruit linked to SBW adults was observed, combined with a prolonged adult activity until the autumn. In this new scenario, we re-investigated SBW biology, ecology, monitoring tools, and potential control methods to develop Integrated Pest Management (IPM) strategies. Several trials were conducted on strawberry in the laboratory, field and semi-natural habitats. The feeding activity of adult SBW results in small deep holes on berries at different stages, causing yield losses of up to 60%. We observed a prolonged survival of newly emerged adults (>240 days) along with their ability to sever flower buds without laying eggs inside them in the same year (one generation per year). SBW adults were present in the strawberry field year-round, with movement between crop and no crop habitats, underlying a potential role of other host/feeding plants to support its populations. Yellow sticky traps combined with synthetic attractants proved promising for both adult monitoring and mass trapping. Regarding control, adhesive tapes and mass trapping using green bucket pheromone traps gave unsatisfactory results, while the high temperatures provided by the black fabric, the periodic removal of severed buds or adults and Chlorpyrifos-methyl application constrained population build-up. The findings are important for the development of an IPM strategy.

## 1. Introduction

The strawberry blossom weevil (SBW), *Anthonomus rubi* Herbst (Coleoptera: Curculionidae), is a serious pest of soft fruit including strawberry (*Fragaria* × *ananassa*), raspberry (*Rubus idaeus*), and blackberry (*Rubus fruticosus*) throughout Europe [1]. On strawberry, this pest can cause high yield losses, often up to 60% [1,2,3,4,5]. The damage is caused by the females, which sever flower buds, hampering flower and fruit development [5,6].

SBW is well documented in the literature as a univoltine species [4,5,7]. Overwintered adults appear in fields in April–May [7] when temperatures reach between 8–15 °C [8,9], where they undergo a feeding period before mating. They feed by drilling small holes through young strawberry leaves and stalks (petioles), with the latter sometimes being completely severed [10]. Later, when flowers are available, they also feed on open flower petals and pollen [1]. Following mating, the female lays eggs in unopened flower buds and then partially or completely severs the peduncles to facilitate the development of the laid egg, normally one per bud [8,10]. The severed buds, once withered, either remain attached to the stalk or completely detach and fall to the ground. However, the stalk is not always severed after oviposition, and therefore, eggs can also be present in non-severed buds. Aasen et al. [11] reported that 40% of undamaged buds contained SBW eggs. In addition, Hellqvist and Winter [12] and Lindblom [13] observed larvae of SBW feeding on pollen in open flowers. Non-severed buds containing an egg develop through to open flowers with a dark spot near the base of the receptacle, resulting in malformed berries [12]. Flowers with such dark spots were commonly observed early in the season in Sweden [12] while only rarely in eastern Norway [11].

Newly emerged adults usually appear in late spring and, after a short period of feeding, enter into summer-winter diapause [14]. It is reported that these adults do not reproduce or sever flower buds and thus cause no economic damage before the following spring [5,7,10,11,15,16]. Over the past few decades in the United Kingdom (UK), due to the large-scale cultivation of growing day-neutral (ever-bearer) cultivars that flower and fruit continuously through summer into the autumn, adults have been observed to remain active, prolonging their life cycle and causing serious damage on flower buds until the autumn [2]. Also, in recent years in some strawberry production, i.e., soilless plantations in plastic tunnels with plants and substrate replaced each year, within the Trento Province in the north-east of Italy, a prolonged presence of damaging adults has been observed into the autumn (Grassi, A. and Miorelli, P. Personal Observation). In addition, in these plantations, new damage was found on fruit. This new damage was observed to be linked to SBW adults. Their feeding activity results in small deep holes in the fruit at different stages: from small and green strawberries through to fully ripe fruit. This new damage on both flower buds and fruit was observed from early May through to late October [17,18].

It is proposed that this alteration in its feeding behavior (i.e., fruit damage) may depend upon a series of factors, including the effects of climate change, the manipulated environment of tunnels where the strawberries are grown, and the prevalent adoption of day-neutral strawberry cultivars. These cultivars offer to the pest a prolonged availability of flowers and fruit during the season (from May to October), allowing it to remain active until autumn, as observed in the UK for flower damage [2]. For the past 20 years, strawberry production in Trento Province in Italy has progressively specialized in the cultivation of ever-bearer strawberries to better respond to market demands. This change to ever-bearer cultivars could also favor a further spread of these newly observed problems within this territory with a general increase of damage. Farmers and field technicians in Trento Province have reported damage of up to 60% on strawberry fruit production caused by the new prolonged feeding habits of SBW [18]. In addition, damage on fruit has not only increased product loss but has also increased the time required to pick and sort the fruit.

Several studies have investigated control strategies against SBW (e.g., mass trapping and insecticide application [2,5,19,20,21,22,23,24,25,26]), but these strategies were developed based upon the original behavior of the insect and to the recorded environmental and agronomic conditions (e.g., soil cultivation in Atlantic climatic areas). The control techniques previously used have been reported as ineffective under current production practices. Additionally, the progressive limitation of authorized insecticide active substances requires the need to direct research efforts towards strategies that provide for the integration of multiple alternatives to chemical treatments alone [27]. Consideration of the newly observed feeding behavior of SBW, in combination with its high impact on strawberry and ineffectiveness of the previously adopted management strategies, resulted in the conclusion that improved IPM strategies were needed. Towards this end, a re-investigation into the type and timing of damage, general biology, ecology, and exploration of new potential control methods relevant to the new production practices is required.

The aim of the study was, therefore, to determine the prospects for developing new IPM systems for SBW in a soilless system under a tunnel taking into account the new pest behavior/activity and its resulting damage. The most important components to consider when seeking to devise an IPM approach are: (1) correct attribution of the damage to the pest; (2) knowledge of pest biology and ecology; (3) reliable monitoring tools; and (4) effective and integrated agricultural, biotechnological, biological, and chemical pest control methods [28].

## 2. Materials and Methods

### 2.1. Assignment of the New Damage on Fruit to SBW and Its Description

In the summer of 2017, several visual surveys were conducted to link this new damage on fruit to SBW adults collected from the strawberry plants. In the field, 10 fruit trusses with 2–3 berries each (cultivar Furore) were caged with an SBW adult for each fruit inside fine net bags. After 10 days, bags were opened, and fruit damage was assessed. In the laboratory, 10 SBW adults were caged (BugDorm-4F3030 Insect Rearing Cage, MegaView Science Co., Ltd., Taiwan) with 3 strawberry fruits for 48 h. This set-up was replicated 4 times. In both situations, the fruit was tested at three developmental stages: small-green, white, and pink/red (ready for harvest). At the same time, we focused our attention to describe this new damage and differentiate it from other biotic and abiotic factors that are well documented within the literature [29]. In addition, 35 specimens collected within and around the strawberry fields were genetically identified at the Department of Agronomy, Food, Natural resources, Animals, and Environment (DAFNAE) of the University of Padua using the barcoding region (mtCOI) following the methodology described by Martinez-Sañudo et al. [30].

### 2.2. Biology of the Pest

We collected severed buds from strawberry fields and in the surrounding semi-natural areas from different host plants (strawberry, wild and cultivated blackberry, *Rosa canina*, and ornamental rose) at different times, dependent upon their presence from May to October in both 2019 and 2020. This information was used to estimate: (1) ability of the pest to infest different host plants, (2) time to develop from egg to adults (2019 only), and (3) rate of emergence. In addition, we assessed if one or more eggs were laid inside each bud. In total, we collected 2708 severed buds in 2019 and 3948 in 2020 (see Supplementary Materials for details).

Newly emerged adults, obtained from buds collected in 2019, were used to verify their ability to damage flower buds and fruit and their egg-laying potential during the same year. Despite information reported in the literature in regard to the lifecycle of SBW [4,5,7], we suspected that SBW in the manipulated environment (soilless, ever-bearing strawberry, nylon protection) could complete more than one generation per year since we found eggs inside severed buds in late October. At the same time, we studied their survival rate under laboratory conditions (see Supplementary Materials for details).

### 2.3. SBW Ecology in a Strawberry Field and the Surrounding Habitat

From February 2019 to October 2020, we carried out observations in and around a strawberry field in Drena (480 m a.s.l.; 45°57’49″ N, 10°57′13″ E; grass cover, soilless, and nylon protected; cultivar Furore; field surface 0.46 hectares; see Supplementary Materials for strawberry field and surrounding habitat description; Figure S1). Our observations on strawberry plants were performed twice a month, from early May to the end of October, to assess the presence and magnitude of the damage caused by SBW. Additionally, we searched severed buds on wild species (herbaceous plants and shrubs) in semi-natural areas adjacent to the field, and adults in potential refuge areas and on weed flowers (flowers of some Asteraceae species are known to be a feeding plant for SBW [31]). The presence of severed buds and adults was assessed both by visual observation of the vegetation and by a tapping method.

In 2019, the presence of adults and severed buds was verified and quantified by manually shaking the vegetation above white support (tapping technique, adapted from Aasen & Trandem [5]) along a 24 m tray line (6 m per tray line in four adjacent lines; ~196 strawberry plants). In 2020, we again quantified the number of adults and severed buds still caught in the canopy by direct observation on strawberry vegetation along a 24 m tray line (6 m per tray line in two adjacent lines in two different tunnels; ~196 strawberry plants). In addition, we counted the severed buds fallen on the substrate surface and damaged flowers of the respective trays.

To quantify the extent of the fruit damage, we directly observed berries from the earliest stages up to their complete ripening. In 2019, we observed 50 fruits for each of the 3 selected ripening classes: small-green, white, and pink/red (ready for harvest). In 2020, the damage assessments were done on 120 fruits at the white-rose stage (30 fruits for each tray line); we decided to use the white-rose stage as a proxy of fruit infestation because it remains on the plant for a longer period and it is not affected by harvesting operations.

### 2.4. Trap Comparison for SBW Adult Monitoring

From August to October 2019, we carried out 3 open field trials to investigate the efficacy of colored sticky panels in catching SBW adults. Trials were carried out in 2 soilless and nylon protected ever-bearing strawberry fields located in Baselga di Pinè (950 m a.s.l., 46°07′13″ N, 11°14′42″ E and 46°07′10″ N, 11°14′03″ E) during the harvesting period.

In the first trial (from 13 August to 7 September; cultivar Favori; production area 0.68 hectares), we compared 6 colored panels (size 25 × 40 cm): sticky traps (Trampas Cromotropicas of Interfase 3, Buenos Aires, Argentina)—blue, yellow; sticky cardboard panels (Impact Board traps of Russell IPM Ltd., Flintshire, UK)—red, black, and polycarbonate panels that we sprayed on both sides with glue (Vebicolla spray, Vebi Istituto Biochimico s.r.l., Padova, Italy)—white, green. All traps were baited with an aggregation pheromone lure (Russell IPM Ltd., Flintshire, UK), which was fixed with a clip in the middle of the upper margin of the south-facing side. Traps were suspended on a wire about 20 cm apart and 40 cm from the soil surface in a random sequence in each of 3 replications. There was at least a 20-m distance between replicates. The panels were replaced one time during the trial to avoid a loss of attractiveness of the color due to the great number of insects caught or the loss of glue efficiency due to drying. The pheromone lure was not replaced as it is effective for 6–8 weeks. SBW adults caught on each side of the traps were counted weekly and removed. Simple observations concerning the side effects of the traps in catching beneficial or pollinator insects were also recorded.

A second trial aimed to compare green and yellow traps, baited and un-baited, with an aggregation pheromone (11 September to 18 September; cultivar Favori; surface 1.1 hectares). Each of the 3 replicates had 6 traps (3 traps of each colour) spaced 10 m apart with the following treatments: (1) un-baited trap; (2) baited with lure from Agralan Ltd., Swindon, UK; and (3) baited with lure from Russel IPM Ltd. Flintshire, UK. Traps were placed in the production area in a randomized block design where each treatment within a replicate is randomized, and each replicate is a block. The height of the panels from the ground and the distance between them was the same as in the first trial. The adults captured were recorded after 7 h, 31 h, and then 7 days. Neither traps nor dispensers were replaced during the trial.

In the third field trial (18 September–20 October) occurring in the same site as the second trial, green and yellow traps (3 of each) were baited or un-baited (see details in the second trial) and randomly placed within the production area at least 10 m apart from each other in a completely randomized design. This was to definitively assess the efficacy of each treatment without possible interferences due to their position in the sequence of each replication. The captures were recorded at weekly intervals for the first two inspections, while the last one was conducted 6 weeks after the traps’ deployment. In all the trials, the traps were placed facing north and south.

We also measured the main color wavelength coordinates (CIE XYZ) of the yellow and green panel model we tested in the field by means of a portable spectrophotometer (Konica Minolta CM-2600D, Konica Minolta Sensing Europe B.V, Milano, Italy).

### 2.5. Pest Control Methods

#### 2.5.1. MASS TRAPPING

Following suggestions from the literature [19,20,21,22,23,24], we tested the mass trapping technique using the commercially available option (green bucket trap with white or green angled cross-vanes (10 cm high, Agralan Ltd., Swindon, UK) baited with a synthetic attractant (Agralan Ltd., Swindon, UK). Each trap contained 150 mL of water with surfactant (50 mL/hl) to break surface tension and was renewed at each trap collection. We tested this trap in two different strawberry fields characterized by different ground management (grass cover or bare soil). In the Drena field (see Section 2.3 and Supplementary Materials for detail of the field) we positioned at ground level (half-submerged in the soil) 46 traps (with white angled cross-vanes) subdivided into 4 blocks of around 400 m^2^ (considered as replications). Another 4 blocks were used as controls. Traps were installed and baited at the end of April 2019. Traps were checked weekly for the first 6 weeks, then twice each month until the end of July, when the experiment ended.

From 19 April to 17 July 2019, SBW adults and damage on flower buds (by tapping) were monitored twice a month and, when fruit was available (after 7 July), damage on 50 fruit/block of each ripeness category (green, white, and pink/red) were also monitored.

In the Baselga di Pinè field (bare soil, soilless, and nylon protected; cultivar Favori; surface 0.18 hectares), we placed 48 traps in a block of approximately 900 m^2^; the second block of 450 m^2^ was used as a control. Traps positioned randomly had a white cross-vane (2/3) or a green cross-vane (1/3); this permitted us to verify the differences between the two cross colors. The trial started at the end of May 2019 and ended on 13 August 2019. Adults captured inside the traps, damage on flower buds and on fruit were assessed 4 times during this period.

#### 2.5.2. ADHESIVE TAPES

We tested the efficacy of adhesive tapes in capturing SBW adults while climbing on the support poles of the soilless cultivation tray, as in the control of *Otiorhynchus* sp. on olive trees [32,33]. In the Drena field, we fixed 60 adhesive tapes on all metal poles of 4 couple tray lines on 9 April 2019. Adjacent lines were used as controls. Each repetition consisted of a couple of tray lines with ~180 strawberry plants. Tapes were checked after 10 and 40 days, and captured adults were counted and removed. On 17 May, adult populations and flower bud damage were both assessed by tapping the canopy of all plants present in the 4 couple tray lines.

#### 2.5.3. GROUNDCOVER

As SBW can readily move among crops to locate a suitable host at the most susceptible developmental stage, we decided to investigate a method to reduce the new adult population in the blackberry crop adjacent to the strawberry crop. This technique could also be used to reduce the population in the crop where it is applied.

The study site was a blackberry field of 1400 m^2^, located in Drena, near the strawberry fields; plants (3 years old, cultivar Lochness) were cultivated in soil (2.2 m between rows and 0.9 m within the row) under nylon tunnels. The soil was partially covered with a black polypropylene woven fabric (60 cm on each side of the row), while the remaining surface was covered by spontaneous herbaceous vegetation. The blackberry canopy extended 30 cm on each side of the row, less than the black fabric width. Plant rows were oriented, allowing sun exposure on both row sides. We hypothesized that the temperature of the black fabric on sunny days could have a negative impact on the development of pre-imaginal stages of SBW inside the severed buds on its surface.

In 2019, during the flowering period, severed buds were collected directly from the blackberry plants and above the groundcover (30 and 76 buds, respectively) as a preliminary investigation. These samples were maintained for 1 month under laboratory conditions and checked for adult emergence or larval mortality.

In the spring of 2020, we collected severed buds from plants and on the black groundcover fabric on 29 May (80 and 173 buds, respectively) and 18 July (63 and 68 buds, respectively). In addition, we deployed severed buds inside fine net bags (15*20 cm; clear organza fabric) on both fabric and in the ground vegetation, the latter acting as a control. We placed inside each bag 20 severed green fresh buds (collected from the plant canopy); 3 bags for each condition were deployed on 29 May, while 1 bag for each condition was deployed on 18 June. After 30 days (on 29 June and 18 July, respectively), bags were removed, and buds were opened to assess the presence and vitality of immature stages or adult emergence holes. To record the temperature, we positioned one data logger inside the bag on each of the two types of surfaces, and another one was suspended 2m high inside the canopy (Elitech USB Temperature Data Logger RC-5, record interval: 15 min).

#### 2.5.4. SEVERED FLOWER BUD REMOVAL

Considering the role of severed flower buds in the build-up of SBW populations, we tested if periodic removal of these buds would reduce pest pressure over time. Trials were performed in the Drena field.

From August to October 2019, we manually and meticulously removed all severed buds, both on the plant canopy and those fallen on the trays or, potentially, onto the ground. To intercept and remove the severed flower buds that would have fallen on the ground, we set up a fine net hammock under the tray line structure but above the grass level. Its shape was decided with consideration of the spatial arrangement of plant canopy and to limit the disturbance to harvesting and agronomic practices (Figure S2). Based on pest biology (time to develop from egg to adult), we decided to remove severed buds every 2 to 3 weeks. The experiment consisted of 4 replications of 4 trays (16 plants) each. Adjacent trays without hammocks were used as control plots; in these control plots, severed buds were observed on plants and trays, counted and left in their position. Severed flower buds and fruit damage were assessed 5 times between 6 August and 4 October. We also noted where these buds were located: plant canopy, tray, or ground.

In the spring–summer of 2020 (from 19 May to 25 August), we improved the methodology to facilitate the removal of severed flower buds. We built “raceways” in 8 strawberry tray lines with an additional 4 tray lines acting as controls; each tray line contained ~100 plants. Test and control tray lines were located in different tunnels, which were physically separated using fine nets along the contact wall. Raceways were made with black polypropylene woven fabric (4 tray lines) or white insect-proof net (4 lines) and held up by U-shaped metallic supports (Figure S3). On half of the tray lines, we periodically removed all the severed buds fallen on raceways using a portable vacuum (DEWALT^®^ mod. DCV517) over intervals of less than 3 weeks. On the other 4 lines, we simply counted the intercepted buds without removing them. Therefore, in the non-removal raceways, we studied the effect of temperature on the mortality rate of pre-imaginal stages on the two different materials. To measure the temperature, we deployed 3 data loggers: 1 on each type of surface and 1 in the grass on the ground surface (Elitech USB Temperature Data Logger RC-5, record interval: 1 min). Along each tray line (*n* = 12), we assessed the number of severed buds fallen on the raceways, infestation rate of these buds, condition of larvae or pupae inside the buds, number of severed buds fallen on the tray substrate surface, and finally the percentage of damaged white strawberry fruit (30 berries/tray line). Checks were performed twice a month until 25 August.

#### 2.5.5. ADULT REMOVAL

Using the experimental plots of the previous trial, on 26 August, we beat the vegetation in the 8 tray lines (26 m total length) to allow SBW adults to fall on the raceways. Fallen adults were counted and then carefully removed. Control plots of the previous experiment remained untreated also in this trial. In the central part of each line (*n* = 12, 32 plants per line), we assessed the number of severed buds fallen on the tray substrate surface, the percentage of damaged white-rose strawberry fruit (30 berries/tray line), and the abundance of flowers with damaged petals (holes due to feeding activity). Checks were performed after 20, 34, and 50 days following adult removal.

#### 2.5.6. INSECTICIDES

In September 2018, the effectiveness of 15 insecticides (Table 1) was compared at Fondazione Edmund Mach in Pergine Valsugana (TN), according to the “Integrated production regulation—IPM of strawberry, small fruits and cherry 2018” of Trento Province (http://www.trentinoagricoltura.it/Trentino-Agricoltura/Disciplinare-produzione-2018, accessed on 30 August 2019).

Each formulation was used at the maximum label dose indicated for strawberry and added with surfactant. Its effectiveness was evaluated by direct contact, manually spraying the solutions on adults previously collected from the strawberry field of Drena. A water plus surfactant (Etravon of Syngenta) treatment was included as a control. We used 100 mL HDPE plastic hand-sprayers (Octopus^®^ Pack), one sprayer for each tested insecticide. The group of 10 adults was sprayed with approximately 2–3 mL solution from a distance of 40 cm. After the treatment, adults were transferred to aerated containers with an untreated strawberry flower truss to provide a feeding substrate. These were maintained under laboratory conditions at 23 °C and 70% ± 10 R.H. Mortality was assessed 24 and 48 h after treatment by visual observation, counting and removing the dead individuals as necessary.

### 2.6. Statistical Analyses

For the developmental time of pre-imaginal stages inside severed buds, a three-parameter sigmoidal equation [y = a/(1 + e−(x − x0)/b)] was run to plot the time-dependent emergence rate curves. Regressions and related statistical analyses were performed and plotted with the software SigmaPlot 11.0 (SPSS, Inc., Point Richmond, CA, USA).

For the survival analysis, we created the survival curves with the function “survfit” using the response variable calculated with the function “Surv” from the package “survival” [34,35] and then plotted with the function “ggsurvplot” from the package “survminer” [36].

For trap comparison, linear mixed-effects models (LME) were used to test the effect of trap color and the presence of dispensers on SBW catches. When necessary, trap data were log-transformed to improve linearity. In each model, color and presence or type of dispenser were entered as categorical fixed factors. Along with the main effects, interactions were also tested. To account for the nested design and repeated measures, block and trap ID were included as random factors. The analyses were performed using the functions “lme” and “anova” from the package “nlme” [37]. The assumptions of the models were evaluated by inspecting diagnostic plots of model residuals (function “qqPlot” of library “car” [38]). The means were compared using the Tukey-HSD test following ANOVA (function “glht” of library “multcomp” [39]).

For mass trapping, adhesive tapes, black groundcover, and periodic removal of the severed flower bud trials, linear mixed-effects models (LME) were used to test the effect of each treatment on SBW abundance and/or pest damage. When necessary, SBW abundance data were log-transformed to improve linearity. Percentage data of fruit damage were arcsine square root transformed prior to analysis. In each model, treatment and time were entered as categorical fixed factors. Again, along with the main effects, interactions were also tested. To account for the nested design and repeated measures, block and plot or trap ID were included as random factors. The analyses were performed using the functions “lme” and “anova” from the package “nlme” [37]. For funnel traps, considering the very low number of catches and the abundance of zeros, a general linear model (GLM) with Poisson distribution was used (function “glm” of library “stats” [40]). The assumptions of the models were evaluated by inspecting diagnostic plots of model residuals (function “qqPlot” of library “car” [38]).

For insecticides, a 2-sample test for equality of proportions with continuity correction (function “prop.test” of library “stats” [40]) was performed using the dead insects at 48 h of each of the 15 insecticides tested against the ones of the control.

With the exception of larval developmental time, all analyses and visualizations were implemented in R 3.4.1 [41].

## 3. Results

### 3.1. Assignment of the New Damage on Fruit to SBW and Its Description

**Figure 1:**
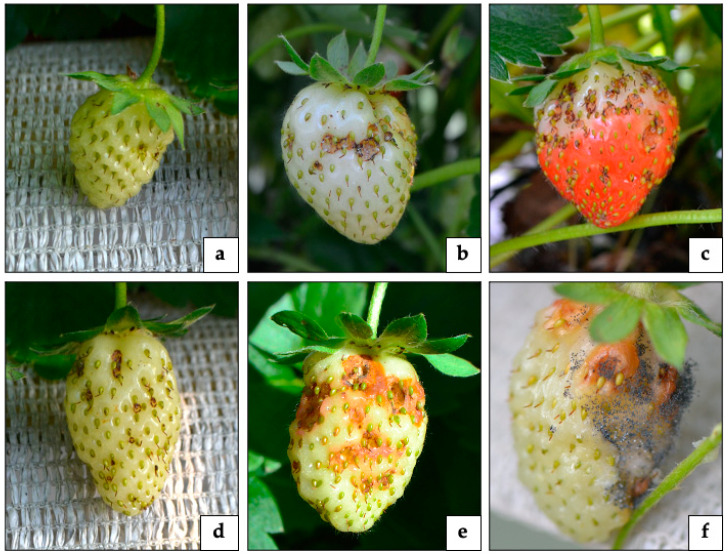
Damage caused by SBW adults on strawberry fruit at different stages: (**a**) small and green, (**b**) white, and (**c**) red strawberry; (**d**) immature malformed strawberry; (**e**,**f**) development of molds on damaged fruit (Tonina-Zanettin©).

Observations in both field and laboratory found SBW adults to cause a previously undescribed type of damage to strawberry. The feeding activity of adult SBW results in small deep holes in the fruit (1.5–5 mm of width and 1–2 mm of depth). This new damage can be caused in fruit at different stages: from small and green strawberries to complete red and ripe berries (Figure 1a–c). When the feeding activity is made on the small and green fruit, the hole can heal, and the berry develops with malformation of its shape (Figure 1d). However, when the damage is done to the white or red fruit, they fail to heal with the consequent development of molds and/or drosophilid flies (Figure 1e,f).

A fragment of the mitochondrial gene COI (the barcode region) of 35 SBW individuals collected within and around the strawberry field was amplified and sequenced, resulting in an approximately 640 bp locus. A comparison of the sequences with GenBank (https://www.ncbi.nlm.nih.gov, accessed on 31 March 2020) and Bold System (www.boldsystems.org, accessed on 31 March 2020) databases showed a similarity > 99% with the species *A. rubi* (Martinez, I. and Mazzon, L. University of Padua, Personal Communication).

### 3.2. Biology of the Pest

From our observations, SBW was able to infest and sever buds of strawberry, cultivated and wild blackberry, *R. canina*, and ornamental roses. Strawberry severed flower buds resulted in less infestation in spring than in summer: 22 ± 16% vs. 38 ± 25% in 2019 (F_1:31_ = 5.84, *p* = 0.02) and 29 ± 26% vs. 62 ± 11% in 2020 (F_1:35_ = 26.31, *p* < 0.001). A variable rate of infestation was observed in the other species: 42 ± 24% and 17 ± 17% in cultivated and wild blackberry, 69 ± 33% for *R. canina*, and 68 ± 29% in ornamental roses. SBW eggs were able to develop to the adult stage in all investigated species.

We determined that usually, one single egg was laid inside each bud (99.9% of buds), but sometimes two or more eggs or larvae were found (6 out of 6656 buds checked in 2019 and 2020; a maximum of 3 individuals/bud). In addition, the presence of holes on the bud sepals did not correspond to the presence of an egg inside, just as the absence of visible holes did not mean that the bud had been severed without laying the egg. During July 2020, we observed SBW infestation in 15% of 92 unsevered buds collected randomly from flower trusses.

We observed a highly variable period of development from egg to adult. Adult emergence was reached in just 17 days after the egg laying in the flower bud but could be up to a maximum of 112 days (see Supplementary Materials and Figure S4).

Newly emerged adults were able to sever the flower buds (172 buds were severed during the trial) but not lay eggs inside them in the same year. In addition, these adults were able to damage strawberry fruit through their feeding activity.

Adults emerged from strawberry, blackberry, and *R. canina* severed flower buds, and adults collected on *Taraxacum* sp. flowers survived more than 240 days with no significant differences recorded between plant species (*p* = 0.68; Figure S5).

### 3.3. SBW Ecology in a Strawberry Field and the Surrounding Habitat

**Figure 2:**
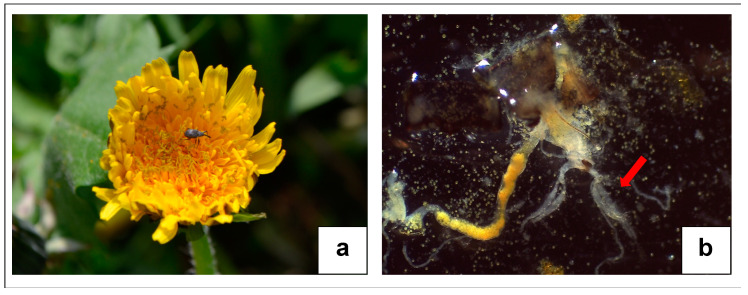
(**a**) Adult of SBW on *Taraxacum* sp. flower (Tonina©); (**b**) *Taraxacum* sp. pollen grains in the insect gut; ovaries (red arrow) were still immature at the end of April 2019 (stereo microscope photo: Grassi©).

At the end of February 2019, some SBW adults were found both inside and in the proximity of the strawberry field: in the crown of *Dactilys* sp. plants present in the groundcover and in moss pads on surrounding dry-stone walls (sheltered areas). No adults were found in litter, empty trays, and field structure components (e.g., poles, nets) during the winter. Nine SBW adults were collected from three crowns of *Rumex acetosa* in the first half of April 2019. In the second half of April 2019, the presence of SBW adults (species confirmed by DNA analysis as outlined previously) was abundant on the copious blossoming of *Taraxacum* sp. spontaneously present in the groundcover inside the tunnels (Figure 2a; Table 2). On dissecting individual adults collected on these flowers, we found *Taraxacum* sp. pollen grains within their gut (Figure 2b and Figure S6).

Following young strawberry transplants being planted inside the tunnels, the presence of SBW adults was observed on them. In early May, in both 2019 and 2020, at the appearance of the first strawberry flower trusses, the first severed buds were observed. Subsequently, the green fruit was damaged and later also the white, red, and ripe fruit.

From mid-May, severed flower buds also appeared on wild and cultivated blackberry, *R. canina*, and ornamental roses. *Rosa canina* ended its flowering period after a month (mid-June) while cultivated and wild blackberries prolonged their availability of flower buds until mid and end of July, respectively. While ornamental roses produced flower buds until October, severed buds were only found until the end of August.

**Figure 3:**
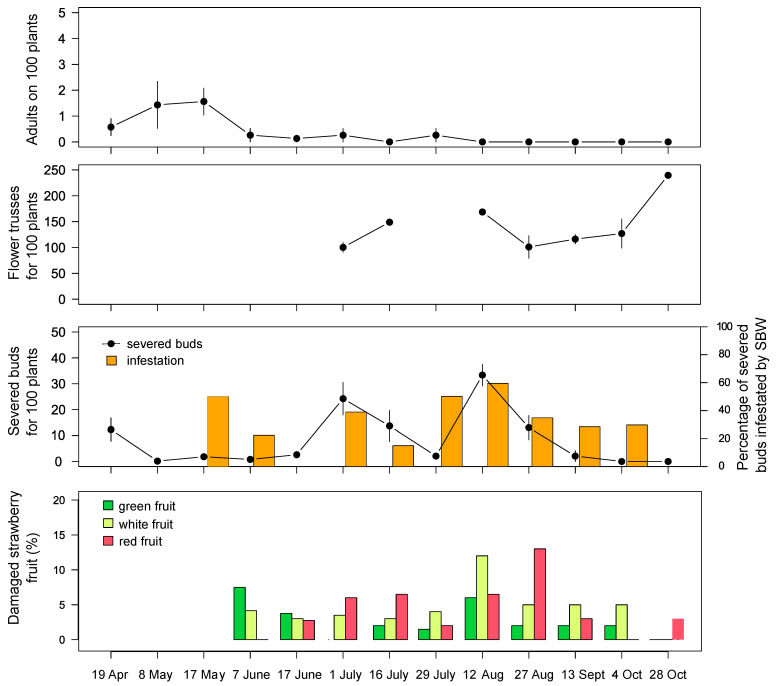
SBW adults, flower trusses, severed strawberry flower buds, percentage of severed buds infested by SBW, and fruit damage during the 2019 season in the field of Drena. Error bars indicate standard error.

**Figure 4:**
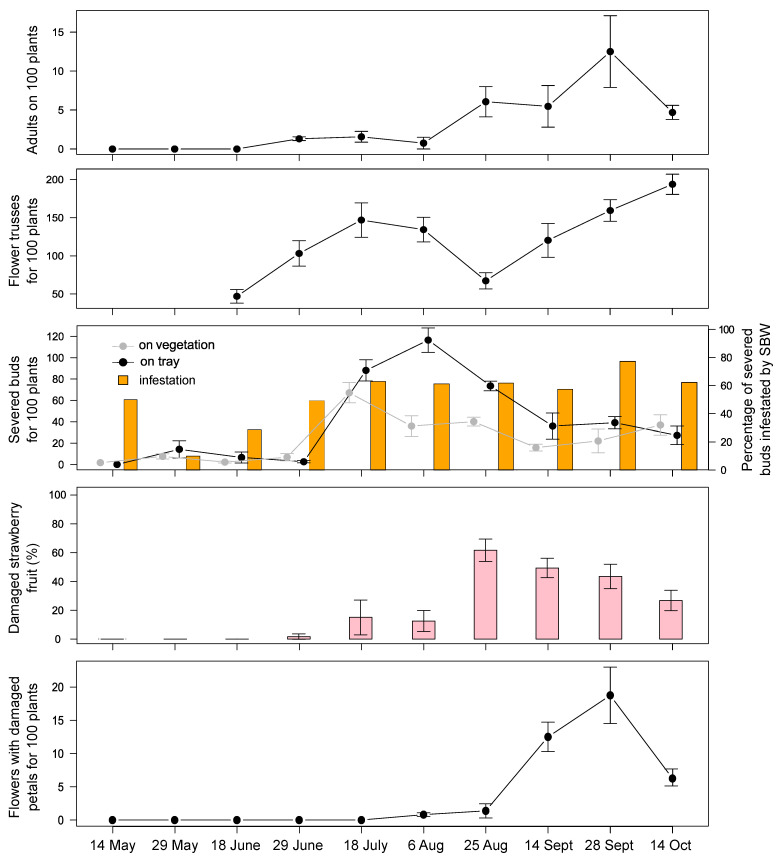
SBW adults, flower trusses, severed strawberry flower buds on vegetation and on tray substrate surface, percentage of infestation, white-rose fruit damage, and flowers with damaged petals during the 2020 season in the fields of Drena. Error bars indicate standard error.

In the field of Drena, in May 2019, ~1.5 SBW adults were observed on 100 plants; subsequently, their abundance decreased to 0–0.3 individuals and remained at this low density throughout the season. Small and green strawberries were damaged in early June (~7%). In mid-August 2019, we recorded the peak damage, with 33 severed buds on 100 plants and 12% of damaged fruit (Figure 3). In 2020, the peak occurrence of severed buds was recorded in early August with low pest pressure. Three weeks later, an increase in adult presence on vegetation was recorded (~6 SBW found on 100 plants), resulting in a strong attack on berries (~60%). In September, damage on flower buds and fruit decreased, while the feeding activity on strawberry flowers increased (highlighted by damaged petals; Figure 4). At the same time, the presence of adults was also recorded on the abundant flowering of *Taraxacum* sp. (1 SBW adult for each 4.3 flowers, 38 flowers/100 m^2^ on October 14). In both years, damage on strawberry buds and fruit was observed throughout the growing season until late October (Figure 3 and Figure 4). During the summer, some adults were found on *Taraxacum* sp., *Ranunculus acris*, and *Senecio inaequidens* (Table 2), of which their flowering was rare due to the frequent mechanical mowing of the grass.

In autumn 2019 and 2020, adults were found on flowers of some herbaceous plants such as *Verbascum blattaria*, *Crepis tectorum*, and again on *S. inaequidens*, and *Taraxacum* sp., present in the spontaneous groundcover inside and on the edge of the strawberry fields (Table 2).

### 3.4. Trap Comparison for SBW Adult Monitoring

**Figure 5:**
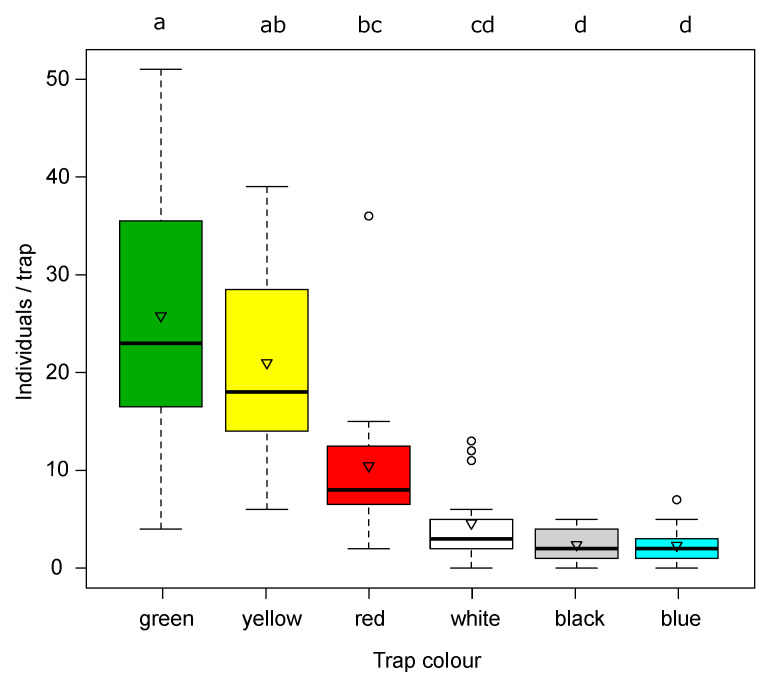
Catches of SBW adults on sticky traps of different colors. The boxes enclose the first and third quartiles; the ends of the whiskers represent the 5th/95th percentiles, the solid lines the median and the triangle the mean. Different letters indicate statistically significant differences between treatments (Tukey’s test following ANOVA, *p* < 0.05).

Among the six colors of sticky traps tested in the first trial, green and yellow traps baited with the Russell pheromone recorded high values of catches (F_5:10_ = 18.96, *p* < 0.001; Figure 5) consistently during the study period (no significant interaction with time F_20:48_ = 1.22, *p* = 0.28). As expected, the yellow sticky panels caught a higher number of Hymenopteran parasitoids compared to the other colors. Honeybees were also observed in large numbers on blue and white traps.

In the second trial, where traps were suspended 20 cm apart, yellow traps caught twice the number of SBW adults than green ones (average 22.4 vs. 10.8 individuals/trap; F_1:10_ = 47.86, *p* < 0.0001) without interaction with the dispenser presence or type (F_2:10_ = 1.03, *p* = 0.39). Even the presence or type of dispenser alone did not give significant effect (F_2:10_ = 0.85, *p* = 0.45).

**Figure 6:**
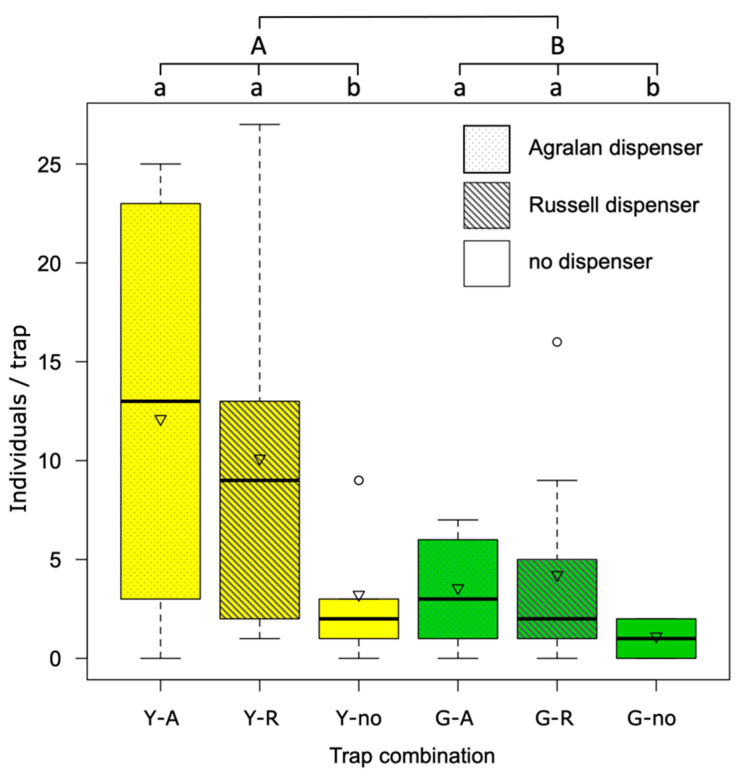
Catches of SBW adults on the yellow (Y) or green (G) sticky traps baited or not with two types of dispensers (A= Agralan, R= Russell, no= no dispenser). The boxes enclose the first and third quartiles; the ends of the whiskers represent the 5th/95th percentiles, the solid lines the median and the triangle the mean. Different lowercase letters indicate statistically significant differences between dispensers, while uppercase letters indicate statistically significant differences between colors (Tukey’s test following ANOVA, *p* < 0.05).

In the third trial, where traps were suspended 10 m distance from each other, yellow traps caught three times more SBW adults than green traps (average 0.99 vs 0.36 individuals/trap/day; F_1:50_ = 6.55, *p* = 0.01). In addition, a positive effect of the dispenser was recorded (F_2:50_ = 3.25, *p* < 0.05); traps with Russell or Agralan dispenser recorded three times more catches than traps without dispenser (0.90, 0.86 and 0.27 individuals/trap/day respectively; Figure 6).

The yellow sticky trap was characterized by the colorimetric values (D65) of L* = 81.26, a* = 3.79, b* = 74.03, X = 54.39, Y = 58.95, and Z = 11.03. In comparison, the green panel values were L* = 38.70, a* = −25.37, b* = 11.37, X = 7.07, Y = 10.49, and Z = 7.67.

### 3.5. Pest Control Methods

#### 3.5.1. MASS TRAPPING

**Figure 7:**
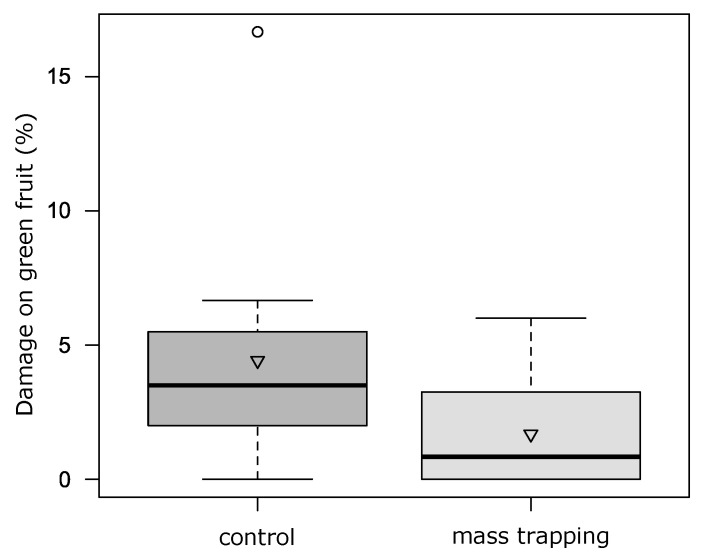
Damage on small green strawberry fruit in control and mass trapping plots (*n* = 12). The boxes enclose the first and third quartiles; the ends of the whiskers represent the 5th/95th percentiles, the solid lines the median and the triangle the mean.

In the field of Drena, over the 12-week period an average of 0.5 ± 1.1 SBW adults/trap/week were caught. On strawberry canopy SBW adult abundance was not influenced by the mass trapping (F_1:5_ = 0.83, *p* = 0.40) over time (interaction treatment*time F_6:29_ = 0.90, *p* = 0.50). No differences were found for the number of strawberry-severed buds (F_1:5_ = 0.79, *p* = 0.41; interaction with time F_6:29_ = 1.28, *p* = 0.29). Damage on small green berries was slightly affected by mass trapping (F_1:5_ = 12.23, *p* = 0.02; interaction with time F_2:12_ = 3.20, *p* = 0.08; Figure 7), while for white and red fruit no differences were recorded (F_1:5_ = 2.58, *p* = 0.17 and F_1:5_ = 0.12, *p* = 0.74, respectively).

In the site of Baselga di Pinè, over the 11-week period an average of 0.4 ± 0.9 SBW adults/trap/week were caught. We found no differences in trap captures between the two cross colors (white vs green; GLM Z = 0.32, *p* = 0.75). The adult abundance on vegetation was not influenced by the mass trapping (F_1:4_ = 0.77, *p* = 0.43) over time (interaction treatment*time F_2:7_ = 0.54, *p* = 0.60). No differences were found for the number of strawberry-severed buds (F_1:4_ = 1.33, *p* = 0.31; interaction with time F_2:7_ = 1.75, *p* = 0.24). For small green, white and red berries no differences were recorded (F_1:4_ = 2.63, *p* = 0.18; F_1:4_ = 0.99, *p* = 0.37 and F_1:4_ = 0.08, *p* = 0.79, respectively).

#### 3.5.2. ADHESIVE TAPES

Adhesive tapes were not effective in capturing adults and preventing damage to strawberry plants. We found only nine adults on the 60 adhesive tapes over 40 days of testing. Adult numbers sampled by tapping the vegetation in mid-May averaged 6.5 ± 4.2 in test plots vs. 7.2 ± 5.2 in control plots without statistical differences (ANOVA after LME F_1:3_ = 0.53, *p* = 0.52). Strawberry severed buds were 7.0 ± 6.9 and 1.7 ± 1.7 in test and control plots, respectively, and not significantly different (ANOVA after LME F_1:3_ = 3.57, *p* = 0.15).

#### 3.5.3. GROUNDCOVER

**Figure 8:**
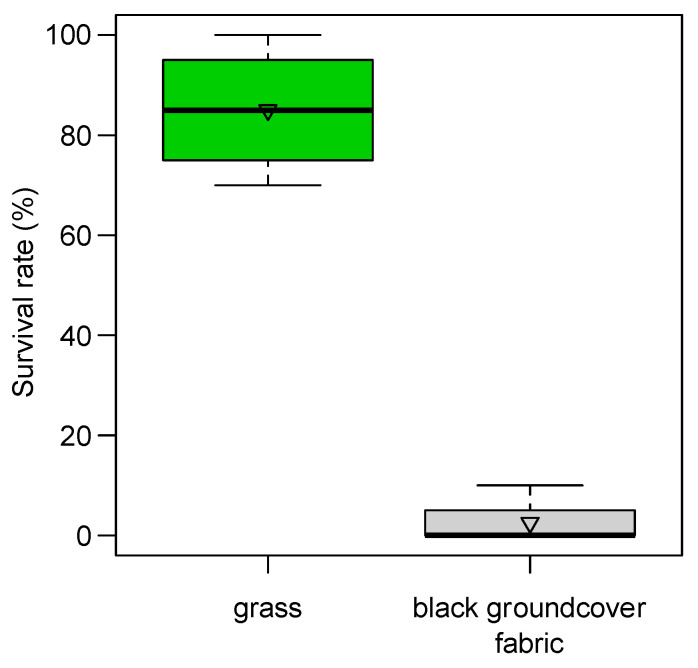
Survival rate of immature stages inside blackberry severed buds deployed on grass (*n* = 4) or on black polypropylene woven groundcover fabric (*n* = 4). The boxes enclose the first and third quartiles; the ends of the whiskers represent the 5th/95th percentiles, the solid lines the median and the triangle the mean.

From the preliminary observation of 2019, we found a reduction in adult emergence from 63% of blackberry severed buds collected directly from plants to 4% of those collected above the black polypropylene woven groundcover fabric. In 2020, severed buds collected on the black groundcover fabric reached 11% of adult emergence, while those collected from plants reached 35% of emergence (68% of corrected mortality). In the standardized trial of 2020, black groundcover fabric was able to strongly reduce the survival rate of immature stages (98% of corrected mortality; ANOVA after LM F_1:6_ = 142.04, *p* < 0.001): severed buds deployed in the grass on the ground showed 86 ± 7% of live stages compared with the 2 ± 2% of those deployed on black fabric (Figure 8).

During sunny periods, the black groundcover fabric frequently exceeded 40 °C (avg. 32.6 °C, with a peak reached around 4pm), reaching a maximal temperature of 54 °C; these temperatures were much higher than those recorded in the grass (avg. 24.4 °C, max 36 °C at 2 pm) and within the blackberry canopy (avg. 25.6 °C, max 33 °C at 2 pm) (Figure S7).

#### 3.5.4. SEVERED FLOWER BUD REMOVAL

**Figure 9:**
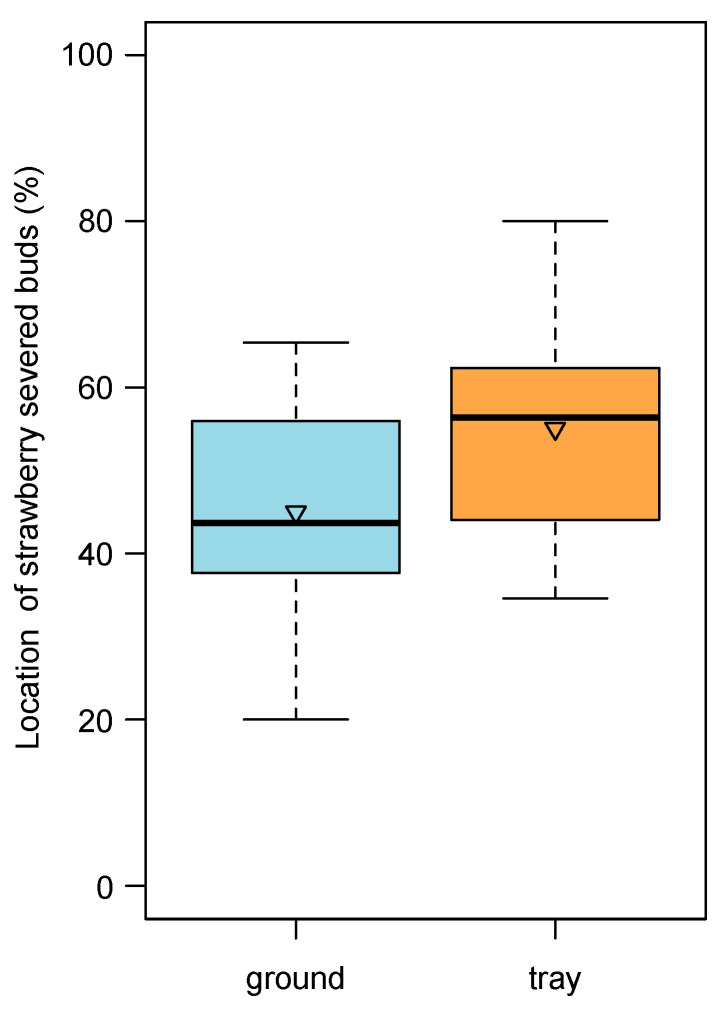
Location of strawberry severed buds between tray substrate surface and ground (intercepted by the hammock). The boxes enclose the first and third quartiles; the ends of the whiskers represent the 5th/95th percentiles, the solid lines the median and the triangle the mean.

Of the total amount of strawberry severed flower buds, we observed that about 55% fell on the tray substrate surface, and the remaining 45% were intercepted by our hammock (under normal conditions, they would have fallen onto the ground; Figure 9).

**Figure 10:**
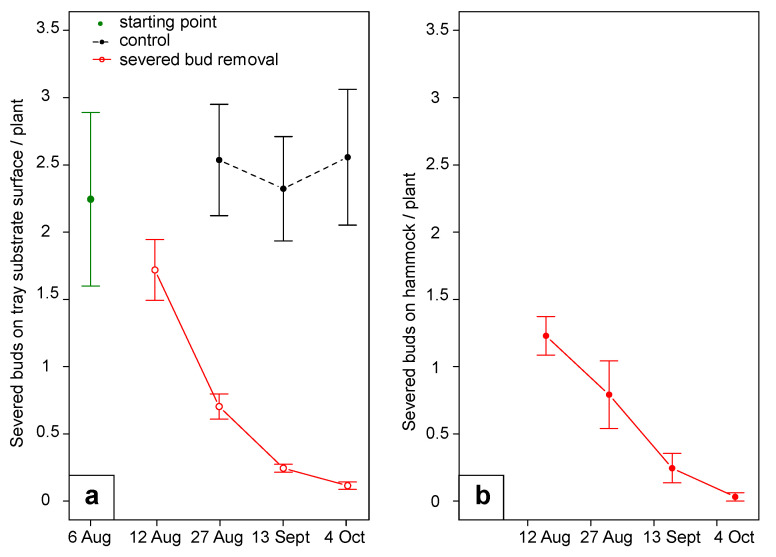
(**a**) Severed buds fallen onto the tray substrate surface and (**b**) intercepted by hammock. Error bars indicate standard error.

The periodic removal of the severed flower buds tested in 2019 significantly reduced the magnitude of the damage on flower buds (F_1:3_ = 25.15, *p* = 0.01). Specifically, severed buds per tray decreased from 9.0 ± 5.2 to 2.8 ± 0.7 severed buds per tray (four strawberry plants per tray) during the first month, reaching 0.5 ± 0.2 severed buds per tray after two months. In the control plots, damage remained constant during the trial at 9.8 ± 3.2 severed buds per tray (Figure 10). Regarding the damage on fruit no differences were observed (F_1:24_ = 0.34, *p* = 0.56; F_1:28_ = 0.51, *p* = 0.48, and F_1:28_ = 0.00, *p* = 0.94 for small green, white and red berries, respectively).

Raceways, used in the summer of 2020 to facilitate the removal of the number of severed flower buds that would have otherwise fallen on the ground, proved ineffective in damage reduction. There were no significant differences between treatments for severed buds on the raceways (F_3:4_ = 1.75, *p* = 0.29; interaction treatment*time F_15:20_ = 0.49, *p* = 0.92). The number of severed buds fallen on the tray substrate surface was not influenced by treatment (F_4:7_ = 1.55, *p* = 0.29); however, a significant interaction between treatment and time was recorded (F_20:35_ = 3.76, *p* < 0.001): in the second half of the trial, severed bud numbers were slightly higher for the white raceways over the black ones. The percentage of damaged white-rose berries was affected by treatment (F_4:7_ = 4.30, *p* = 0.04) without interaction with time (F_20:35_ = 0.84, *p* = 0.65). White raceways without bud removal plots were characterized by greater fruit damage compared to black raceways without bud removal; control and raceways with bud removal plots did not statistically differ from the other treatments.

In 2020, 52 ± 18% of the severed flower buds fell on the tray substrate surface, while the remaining 48 ± 18% were intercepted by the raceways. During full sun hours (from 11am to 5pm), the raceways made with black polypropylene woven fabric or white insect proof net reached temperatures of 35 °C (avg. 30.2 °C) or 30 °C (avg. 27.8 °C), respectively; these temperatures were much higher than those recorded in the grass (avg. 23.6 °C, avg. of maximum daily temp 26 °C) (Figure S8).

The severed buds collected over three-week intervals displayed an infestation rate of 52 ± 23%. Dissection of the infested buds revealed the following: 94.4% active larvae (very small if the bud was still green, bigger if the bud was browned), 2.9% dead larvae, 0.5% alive pupae, and 0.4% alive newly formed adults not yet emerged. Only 0.5% of brown severed buds presented adult emergence holes, with none from the fresh green ones. The remaining 1.3% of buds were marked by feeding activity but without the presence of an individual (buds probably contained dead larvae at young stages or preyed upon individuals).

#### 3.5.5. ADULT REMOVAL

**Figure 11:**
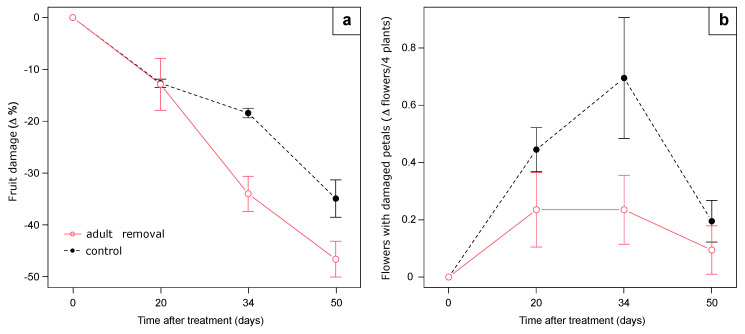
(**a**) Dynamics of fruit damage expressed as change in percentage of damaged fruit from time 0, and (**b**) percentage of flowers with damaged petals, expressed as percent change in flowers per 4 plants. Error bars indicate standard error.

At the beginning of the experiment, on 26 August 2020, 354 adults were counted and removed from the eight lines (on average, 13.6 adults/m of line length). The number of severed buds fallen on the tray substrate surface was not influenced by treatment (F_1:10_ = 0.48, *p* = 0.50) or its interaction with time (F_3:30_ = 0.23, *p* = 0.87). After 34 and 50 days from adult removal, the decrease of the percentage of damaged berries was higher in plots where adults were removed than in the controls (F_1:10_ = 3.77, *p* = 0.08; interaction treatment*time F_3:30_ = 3.26, *p* = 0.03; Figure 11). We found a marginal significant treatment effect on the abundance of flowers with damaged petals (F_1:10_ = 3.65, *p* = 0.08), with a higher presence in control plots than in those with adults removed, especially after 20 and 34 days following treatment.

#### 3.5.6. INSECTICIDES

Our trial found only 1 of the 15 tested insecticides to show efficacy: Reldan LO (a.i. Chlorpyrifos methyl), which caused 100% mortality 48 h after the treatment (with statistical difference compared with the control of *p* < 0.0001). Trebon UP (a.i. Etofenprox) and Epik SL (a.i. Acetamiprid) reported 20% and 10% mortality, respectively (no significant difference compared with the control; *p* = 0.23 and *p* = 0.5, respectively).

## 4. Discussion

### 4.1. Assignment of the New Damage on Fruit to SBW and Its Description

The new damage caused by the feeding activity of SBW adults was carefully described in the results section. This damage is different from that caused by other biotic (e.g., earwigs, wasps, or slugs) and abiotic factors (e.g., lack of pollination), and from the development of un-severed flowers infested by SBW larvae. The damage on fruit, in addition to that on flower buds, increases product loss and the resulting picking and sorting times. Similar damage was also produced on raspberry fruit; here, the tropic activity of SBW resulted in small and circular holes (1–2 mm of width and 1–2 mm of depth). The genetic confirmation of the species causing damage in strawberry was *A. rubi*, removing the potential that a new congeneric species or a forest species was the cause. In the genus *Anthonomus*, there are species within which newly emerged adults can also perforate the fruit for feeding activity before overwintering (e.g., *A. pomorum* [33,42]). Therefore, SBW could also carry out this activity.

### 4.2. Biology of the Pest

From our observations, SBW was able to infest and severe flower buds of strawberry, wild and cultivated blackberry, *R. canina*, and ornamental roses. All these species are already reported as host plants [1,7,32,33,43,44], and SBW is well known to also feed on foliage and flower buds of other Rosaceae plants [1,7,32,33,44].

We observed the presence of laid eggs in ~35% of strawberry severed buds in 2019, while 58% of severed buds were infested in 2020. These values are lower than those reported in the literature [10,11], probably because a huge amount of buds was also severed by newly emerged adults, which can damage flower buds without laying eggs inside them in the growing season. However, some authors reported that newly emerged adults do not sever buds, and therefore, they are not considered able to cause any economic damage until the following spring [5,7,10,11,15,16]. In addition, the newly emerged adults observed here were able to damage strawberry fruit through their feeding activity.

As reported in the literature [1,11,45], SBW completes its pre-imaginal development on average in 30–40 days. However, in our trials, adult emergence was observed in as few as 17 days after the egg laying in the flower bud and up to a maximum of 112 days; this information should be taken into account for the implementation of any pest control strategy. Different developmental time was found among host plants and for strawberry between severing period (spring or summer). Potentially the longer time that eggs inside summer severed buds need to achieve the adult stage may have positive implications in regard to overwintering aspects. The prolonged survival of newly emerged adults and their inability to lay eggs allow us to also confirm that SBW performs only one generation per year in a manipulated environment, such as that of the strawberry tunnels. In addition, a prolonged presence of eggs in severed buds (from May to October) corresponds a protracted emergence of the new adults. Adults emerging in late summer may easily overwinter and therefore have better all-round performance in the following strawberry growing season. This aspect of species biology deserves to be further investigated.

### 4.3. SBW Ecology in a Strawberry Field and the Surrounding Habitat

We observed the presence of SBW adults inside the strawberry field during all four seasons. The manipulated environment of growing tunnels can offer an overwintering site since we found adults at the end of the winter period (February) in sheltered areas inside and close to the field (e.g., crown of weeds, moss pads). This finding is in accordance with other studies which found SBW adults inside strawberry fields [11,46,47] as well as in open fields [8,10,11,48]. In fact, Lindblom [13] reported grass as an overwintering site for SBW adults.

In early spring (April), when strawberry plants are not yet present inside the field, the abundant presence of adults on the copious blossoming of *Taraxacum* sp. supports the hypothesis that feeding on this and other plants could provide food resources to overwintered adults at the beginning of the season and therefore favor the maturation of their eggs. In fact, we found *Taraxacum* sp. pollen grains in the gut of adults collected from these flowers. During the strawberry growing season (from May to October), the low presence of SBW adults on wildflowers could be related to: the frequent mowing of the grass groundcover that, therefore, offers a poor food source since blossoms are rare; abundant strawberry flowers may be more attractive for the insect; or wildflowers may have a feeding role only in early spring (egg maturation) and late summer–autumn (an energy source for the overwintering period). In fact, in autumn, adults were more abundant on a higher number of herbaceous flower species. The possible role of these plants in the preparation of the overwintering period should be investigated to implement IPM strategies that could consider habitat manipulation practices such as frequent grass mowing. It is worth noting that SBW adults were found exclusively on yellow flowers of herbaceous plants. In this regard, Balachowsky and Mesnil [31] reported the presence of SBW adults on some herbaceous Asteraceae species, such as *Tragopogon pratensis*. So not surprisingly, the scientific name of the SBW genus derives from Greek: anthonomos = feeding on flowers, from ánthos (flower) + nomos (to pasture) [49].

Focusing on the strawberry field, when the young transplants are planted inside the tunnels (around the end of April), they are promptly visited by SBW adults who initially feed on flower buds, open flowers, petioles, and thereupon also on strawberry fruit. The first strawberry severed buds appear immediately after the development of the first flower trusses. After a couple of weeks, SBW start damaging green fruit and later also white and red-ripe fruit. Damage on both flower buds and fruit can be observed throughout all the growing season, from May until late October, with the highest magnitude in August in both years of investigation. In the second year of investigation, the increase in fruit damage clearly emerged when the amount of available flower trusses decreases, suggesting a shift of the pest from flower buds to berries. Later in the growing season, damage on flower buds and fruit decreased, while the SBW feeding activity on strawberry open flowers (highlighted by damaged petals) and on *Taraxacum* sp. flowers suggests its increasing interest in pollen nutrition.

It is worth underlining that the phenology data were collected in a commercial strawberry field; therefore, the drop of SBW adult presence in late May 2019 can be related to the insecticidal treatment with chlorpyrifos-methyl performed on 14th May, after the first strawberry flowering period. Differently, in 2020 a treatment with the same insecticide was applied earlier in the season on young, transplanted plants (14 April); this can explain the low population density observed during spring 2020 (May–June).

We observed that a small number of adults on vegetation seems to be sufficient to produce significant damage on fruit. Currently, since this new damage on fruit has only been recently recorded, we are not yet able to fix a threshold for damage on fruit. This aspect should be further investigated.

### 4.4. Trap Comparison for SBW Adult Monitoring

Among the six colors of sticky traps baited with the Russel dispenser tested, green and yellow traps recorded higher values of catches of SBW adults than red, white, black, and blue traps.

When traps were closely positioned (20 cm from each other), yellow traps caught more SBW adults than green ones regardless of the presence or not of the dispensers. The proximity of the traps probably generated a cloud of attractant, which was presumably equally effective for any of the traps. In this way, the high attractiveness of the yellow color was clearly demonstrated. Differently, when the traps were 10 m from each other, the positive effect of both dispensers was demonstrated.

We confirmed the high attractiveness of yellow traps combined with the synthetic attractant. Our results on trap color confirm data reported by Jay et al. [6], where yellow sticky traps, although without the synthetic attractant, resulted in being more effective than other tested colors (blue, white, and transparent). Our results confirm the efficacy of the dispenser containing the male aggregation pheromone highlighted in other studies [19,50,51,52,53]. From our observations (Grassi, A. and Puppato, S. Personal Observation), the type of yellow is important in determining trap attractiveness; in the results section, we provided the colorimetric values. This aspect should be considered when traps are selected. It is worth noting how the most attractive color for traps is yellow, the same color of wildflowers on which we found SBW adults feeding on their pollen.

The combination of the yellow sticky trap with the synthetic attractant can be promising for both adult monitoring and mass trapping; further studies should take into account the collateral effects on beneficials. In fact, especially for mass trapping implementation, it is fundamental to study the adverse effects upon useful insects and arthropods in light of the strong attractiveness of yellow sticky traps [54,55,56]. In this context, the green color could provide acceptable SBW catches and reduce impact upon beneficials.

### 4.5. Pest Control Methods

#### 4.5.1. Mass Trapping

We used the green bucket trap currently available on the market, with white or green angled cross-vanes, as suggested by Baroffio et al. [24], combined with the synthetic attractant as recommended by Fountain et al. [22], Wibe et al. [53] and Baroffio et al. [19]. Traps were positioned in the field early in the season (April) to intercept the overwintered adults, as recommended by Baroffio et al. [24]. Unlike the positive results obtained by Baroffio et al. [24], who found a significant reduction of the damage, under our conditions, the traps were not effective for either adult monitoring or mass trapping. In fact, despite the presence of SBW adults on strawberry plants and relative damage on flower buds and fruit, catches were rare. Baroffio et al. [19] report the low efficacy of traps in capturing insects attracted to the traps as adults can escape from them; therefore, this aspect could explain the lack of efficacy we found. Therefore, for mass trapping, the use of the synthetic attractant could be more promising if combined with sticky traps, which prevent the insect from escaping.

#### 4.5.2. Adhesive Tapes

Adhesive tapes applied to tray poles showed no efficacy in capturing adults and preventing damage on strawberry plants. Flying ability was reported by Jary [10], and we also observed this in both the laboratory and field conditions. Therefore, this explains the lack of efficacy of this technique.

#### 4.5.3. Ground Cover

The black polypropylene woven groundcover fabric hampered the development of the pre-imaginal stages sheltering inside the blackberry severed buds. This increase in mortality was probably caused by the high temperatures reached on the black surface of the fabric (more than 50 °C in the hottest hours, about 20 °C warmer than the grass surface). For an advantageous effect in the use of black fabric groundcover, its width must be related to the plant canopy in order to catch all buds that fall from it. When different crops attractive to SBW are closely cultivated, it becomes fundamental to have a wide area management strategy, especially with species that bear flower buds before others. In this context, severed bud management with black groundcover fabric in blackberry fields, a crop that flowers once a year lasting a month between May and June, can have positive effects on neighboring crops of day-neutral or late short-day strawberry. This could result in a decrease in the newly emerged SBW adult population and consequent migration from one crop to an adjacent crop.

#### 4.5.4. Severed Flower Bud Removal

In late summer 2019, the periodic and precise removal of all the severed buds significantly reduced the damage on strawberry flower buds; this could be related to the decrease of newly emerged adults. Although no statistical differences regarding the damage on berries were observed as a result of severed bud removal, a slight reduction in fruit damage was recorded on green small berries. However, this unsatisfactory result could be explained by the great ability of SBW adults in damaging berries, also at low population densities. In this preliminary trial, treated and control plots were close to each other, and therefore, some adults could have had the possibility to move to adjacent fruit. Following these considerations, in 2020, we investigated the damage dynamic using the entire tray line as plots and starting from the beginning of the growing season (May). In addition, it was desirable to individualize a technique that allows optimizing the complete removal of the severed buds both in terms of time and costs. Nevertheless, the removal of severed flower buds that fall on the tray substrate surface appeared difficult to realize and therefore, merely the removal of severed flower buds that otherwise would have fallen on the ground was tested using raceways to facilitate their removal. Even though nearly half of the severed buds were intercepted by the raceways, the technique was not effective to reduce damage to buds and fruit. Considering our results, we believe that the management of even half of the severed buds is not enough; the remaining infested buds can itself boost the newly emerged adult population. Therefore, to effectively control the pest population and its relative damage, even the severed buds which fall on the tray substrate surface must be removed. In no bud removal plots with white insect proof net raceways, damage on both flower buds and fruit reached the highest values. This unsatisfactory result could be related, on the one hand, to the above-mentioned low efficacy of only partial removal of severed buds and, on the other hand, to the low mortality of immature stages, which were able to complete their development inside buds that fell on the white surface. In fact, as white raceways recorded cooler temperatures than black ones, they probably allowed recovery of favorable conditions during the night. In addition, newly emerged adults can reach flower and fruit trusses easily due to a short distance in respect of those emerged at ground level (which are also probably exposed to their natural enemies). In bud removal plots, pest population build-up was constrained throughout the periodic severed bud collection over a three-week interval. In fact, only 0.25% of severed buds allowed adult emergence within this period. In black plots, no differences were observed between severed bud removal and no removal plots since the temperatures reached by the polypropylene woven fabric could have had a marginal role in reducing immature stage development. In this experiment, the recorded temperatures (avg. 30.2 °C) were lower than expected (compared to those recorded in the groundcover trial from end-May to mid-July, avg. 32.6 °C), probably due to the installation of a shading screen above the tunnels by the farmer during the summer. To implement this technique, it should be considered that the removal of the severed flower buds must occur at maximum intervals of three weeks in order to hamper the development to the adult stage. Finally, to effectively reduce the damage on flower buds and fruit, the severed bud removal should be undertaken early and be continuous throughout the growing season because we observed that SBW females are able to lay eggs from early May to the end of October.

#### 4.5.5. Adult Removal

The removal of adults when the maximum amount of damage on flower buds and fruit was recorded resulted in the removal of a considerable number of individuals (~1.7 adults/plant) and consequently decreased the pest pressure. The decrease in fruit damage was observed 34 days after adult removal; after 20 days, no differences were observed because this sampling time still considered berries that started their development before the beginning of the trial. However, after 20 days from the removal, the positive effect of population suppression emerged by the slight difference of flowers with damaged petals since they are affected by SBW feeding activity in autumn. No differences were observed in the number of severed buds fallen on the tray substrate surface, probably because in this period, a decrease in both flower trusses and severed bud abundance was generally observed (see Figure 4). The removal of adults was determined to be a useful technique in reducing damage on fruit; it would be interesting to apply it from the beginning of the growing season when adults start to colonize strawberry plants to evaluate its early effects on pest and damage dynamics. At the same time that we performed our experimental trial, the Drena strawberry grower applied this method in other fields with satisfactory and encouraging results (Miorelli, P. Personal Communication).

#### 4.5.6. Insecticide Evaluation

Among the fifteen insecticides tested in our experiment by direct contact with adults, only the one containing chlorpyrifos-methyl resulted in efficacy for SBW control. Our results are supported by Aasen & Trandem [5] and Blümel [25], who reported that some new pesticides are not very efficient in controlling the pest. In fact, the control of strawberry flower pests is based on broad-spectrum organophosphorus insecticides such as chlorpyrifos [2], which negatively influences the beneficial fauna (e.g., pollinators and natural enemies). In addition, insecticide-based strategies to control SBW are not very effective due to its cryptic behavior (thanatosis) [57] and with its development sheltered within the flower bud. In addition, resistance to insecticides is reported for pyrethroids and is expected to increase in level and distribution [5,25]. The progressive limitation of the availability of plant protection products, such as the non-renewal of the approval of the active substance chlorpyrifos-methyl in accordance with Regulation (EU) 2020/17, should be taken into account. Therefore, the management of this phytophagous pest must integrate production strategies that limit the use of insecticides in order to satisfy the consumer demand for fruits with low residues [26].

## 5. Conclusions

Our observations provide clarification of some of the differences that have arisen within the rich literature as well as described new additional feeding habits displayed by SBW in our specific habitat. Damage on fruit is caused by both overwintered and newly emerged adults. A small number of adults on vegetation is enough to produce significant damage on flower buds and fruit. However, currently, no threshold for damage on fruit exists for this pest. The adoption of a new IPM approach is necessary to effectively control the pest considering the poor effectiveness of available insecticides, their progressive use restrictions and deregistration, and the development of resistance. The application of the mass trapping technique, using the available green bucket traps baited with synthetic attractant, showed unsatisfactory results. Nevertheless, the synthetic attractant combined with yellow or green sticky traps showed a strong capacity in capturing adults as a monitoring tool and therefore offers an upcoming potential use in mass trapping application. Pest population build-up was constrained throughout the periodic removal of severed buds, within an interval of three weeks (according to the developmental time from egg to adult). This technique was effective in reducing damaged buds only if severed buds were totally and continuously removed.

To contain the pest population and its impact on the crop, a rationale and targeted IPM strategy should be implemented in consideration of the integration of the main observations that have emerged from our study: (1) the management of overwintered adults from the beginning of the strawberry growing season; (2) the reduction of newly emerged adults by the periodic removal of all severed buds and/or by taking advantage of the black groundcover effect on temperature; (3) the suppression of adults by their periodic removal from the crop vegetation during the season; (4) the use of yellow or green sticky traps as monitoring tools and the investigation of their possible use for mass trapping (period of exposure, density, position, etc.); and (5) the management of alternative hosts and flowering species (cultivated, wild, and ornamental) in and around the field considering their important role in pest population build-up (e.g., blackberry, roses) and in supporting the overwintering adults (e.g., *Taraxacum* sp. in spring and autumn).

## Figures and Tables

**Table 1:** Insecticides tested against SBW adults.

IRAC MoA Group	Active Ingredient	Trade Name	Formulation	Rate (L^−1^)	Manufacturer
Avermectins (6)	Abamectin	Vertimec^®^ EC	1.84% EC	0.6 mL	Syngenta
Neonicotinoids (4A)	Acetamiprid	Epik^®^ SL	4.67% SL	1.5 mL	Sipcam Italia
Pyrethroids (3A)	Acrinathrin	Rufast^®^ E-FLO	7.0% EW	0.6 mL	Cheminova A/S
UN	Azadirachtin	NeemAzal^®^—T/S	10 g/L EC	3 mL	CBC (Europe) S.r.l.
Organophosphates (1B)	Chlorpyrifos-methyl	Reldan^®^ LO	225 g/L EC	4 mL	DOW-Agrosciences
Pyrethroids (3A)	Deltamethrin	Decis^®^ EVO	2.8% EW	0.5 mL	Bayer CropScience
Avermectins (6)	Emamectin benzoate	Affirm^®^	0.95% WG	1.5 g	Syngenta
Pyrethroids (3A)	Etofenprox	Trebon^®^ UP	30.00% EC	0.5 mL	Sipcam Italia
Neonicotinoids (4A)	Imidacloprid	Kohinor^®^ 200 SL	200 g/L SL	0.75 mL	Adama Italia
Pyrethroids (3A)	lambda-Cyhalothrin	Karate Zeon^®^	9.48% CS	0.15 mL	Syngenta
Pyrethroids (3A)	Pyrethrins	Pyganic^®^ 1.4	12.91 g/L EC	2.5 mL	McLaughlin Gormley King Europe Ltd.
Carbamates (1A)	Pirimicarb	Pirimor^®^ 17.5	17.5/100 g WG	2.8 g	Syngenta
	Potassium salts of fatty acids	Ciopper^®^	479.8 g/L EW	20 mL	Alpha BioPesticides Ltd.
Spinosyns (5)	Spinosad	Laser^®^	480 g/L SC	0.25 mL	DOW-Agrosciences
Pyrethroids (3A)	tau-Fluvalinate	Mavrik^®^ 20 EW	240 g/L EW	0.3 mL	Adama
CONTROL (water + surfactant)					

**Table 2:** Herbaceous (H) and shrub (S) flowering species investigated in and around the strawberry field. Abundance of SBW adults: + rarely, ++ frequently, and +++ always.

Plant Species	Family	Localization	Flowering Period	Abundance
*Acetosella corniculata* (H)	Oxalidaceae	field margin	May	not found
*Achillea millefolium* (H)	Asteraceae	field margin	July	not found
*Anthericum ramosum* (H)	Asparagaceae	field margin	August	not found
*Cornus sanguinea* (S)	Cornaceae	field margin	May	not found
*Crataegus monogyna* (S)	Rosaceae	field margin	May	not found
*Crepis tectorum* (H)	Asteraceae	inside field	September	+
*Ononis natrix* (H)	Fabaceae	field margin	July	not found
*Ranunculus acris* (H)	Ranunculaceae	inside field	August	+
*Rhamnus frangula* (S)	Rhamnaceae	field margin	May	not found
*Robinia pseudoacacia* (S)	Fabaceae	field margin	May–June	not found
*Saponaria ocymoides* (H)	Caryophyllaceae	field margin	July	not found
*Senecio inaequidens* (H)	Asteraceae	field margin	June–October	++
*Silene vulgaris* (H)	Caryophyllaceae	field margin	June	not found
*Taraxacum* sp. (H)	Asteraceae	inside field	April–November	+++
*Verbascum blattaria* (H)	Scrophulariaceae	field margin	July–October	+
*Viburnum lantana* (S)	Caprifoliaceae	field margin	May	not found

## Data Availability

All data can be made available from the lead author upon request.

## Supplementary Materials

The following are available online at https://www.mdpi.com/article/10.3390/insects12080701/s1: Biology of the pest—Materials and Methods; Table S1. Temperatures ± SD recorded in the laboratory for the maintenance of severed buds and adult survival; Description of Drena strawberry fields and surrounding semi-natural areas; Figure S1. Detail of strawberry field in Drena with strawberry soilless cultivation trays arranged in lines, supported by poles; Figure S2. Detail of the hammock set up a fine net under the tray line structure and above the grass level; Figure S3. Detail of the raceways made with black polypropylene woven fabric or white insect proof net and held up by U-shaped metallic supports; Biology of the pest—Results; Figure S4. Time-dependent emergence rate for strawberry buds severed in spring (blue) and summer (red) 2019; Figure S5. Survival analyses for adults from the 4 different plants; Figure S6. *Taraxacum* sp. pollen grains found within the gut of dissected adults collected on these flowers; Figure S7. Average temperature recorded on the grass (green), on black groundcover fabric (grey), and within the blackberry canopy (blue), hourly during the period 29 May–18 July 2020; Figure S8. Average temperature recorded on black polypropylene woven fabric raceway (grey), on white insect proof net raceway (white), and on the grass (green).
