# Supplementary material for: Anthonomus rubi on Strawberry Fruit: Its Biology, Ecology, Damage, and Control from an IPM Perspective
Source: Insects. 2021 Aug 5;12(8):701. doi: 10.3390/insects12080701 (PMC8396509; doi:10.3390/insects12080701)
Supplement: Supplementary file 1 [file insects-12-00701-s001.zip › insects-1318444-supplementaryfinal.pdf]

insects

MDPI

Supplementary Material

Biology of the pest – Materials and Methods

Severed buds were collected from strawberry and other host plants (wild and cultivated blackberry, *Rosa canina* and ornamental rose) available in semi-natural areas surrounding the fields. Samplings were performed twice a month covering areas of 1.000-2.000m$_2$ for the fields. Buds were singularly placed in 2ml plastic tubes closed with cotton and pooled per sample unit into open plastic boxes. Buds were maintained in a chamber with a high level of humidity (~90%), to reduce dehydration, under laboratory temperatures (ranging from ~20°C in winter to ~27°C in summer, as reported in Table S1) and checked weekly for adult emergence. After two to four months, they were dissected under a stereomicroscope and any larvae, pupae or non-emerged adults were counted and recorded.

Table S1. Temperatures ± SD recorded in the laboratory for the maintenance of severed buds and adult survival.

Time	Average Temperature (°C) (±SD)
June 2019	28.1 (± 2.2)
July 2019	27.9 (± 1.6)
August 2019	28.1 (± 1.0)
September 2019	24.9 (± 2.0)
October 2019	20.7 (± 1.5)
November 2019	19.0 (± 0.9)
December 2019	19.3 (± 1.0)
January 2020	18.8 (± 0.6)
February 2020	19.5 (± 0.6)
March 2020	19.0 (± 0.8)
April 2020	20.0 (± 0.9) May 2020 22.8 (± 1.2)
June 2020	25.3 (± 2.0)
July 2020	27.9 (± 1.7)
August 2020	28.5 (± 1.2)
September 2020	25.3 (± 1.7)
October 2020	20.3 (± 1.2)

The adult survival experiment was performed under laboratory conditions (temperatures are reported in Table S1; 60-80% R.H.; 16:8 L:D photoperiod). For this experiment, we used 299 adults that had emerged from strawberry buds, 27 from blackberry buds, and 14 from *R. canina* buds. Also 16 adults collected from *Taraxacum* sp. flowers were observed. Adults (3 to 31 depending on availability of newly emerged adults) were caged in 1L plastic boxes with a ventilated lid. Water supply, one-two strawberry leaves and/or one flower truss and/or one fruit were provided. Adult mortality and severed buds were assessed weekly; at the same time, food supplies were renewed.

Description of Drena strawberry fields and surrounding semi-natural areas

Drena strawberry fields are a soilless and nylon protected cultivation system, with cultivation trays arranged in lines, supported by metallic poles (Figure S1). For each tray, 3 or 4 strawberry plants are transplanted, according to the cultivar.

Grass cover consisted mainly of different wild species of Poaceae, with *Rumex* sp., *Taraxacum* sp., and other flowering species (the investigated flowers are reported in Table 2, main text).

The fields are surrounded by semi-natural areas for most of the perimeter. The surrounding semi-natural areas are mostly coppice forests with trees 10 – 15m tall. The tree species are *Carpinus betulus*, *Castanea sativa*, *Fagus sylvatica*, *Quercus pubescens*, *Fraxinus ornus*, and *Robinia pseudoacacia*. The understorey vegetation included *Cornus mas*, *C. sanguinea*, *Crataegus monogyna*, *Rhamnus frangula*, *Rubus* sp., and *Viburnum lantana*. A ditch with a low wall and waterbased vegetation (e.g. moss pads, *Epilobium* sp.) passes near the field used for most of the experiments. The fields where the “adhesive tapes” experiment was performed borders on one side with the blackberry field and is rich in dry-stone walls with moss pads and wild ferns.

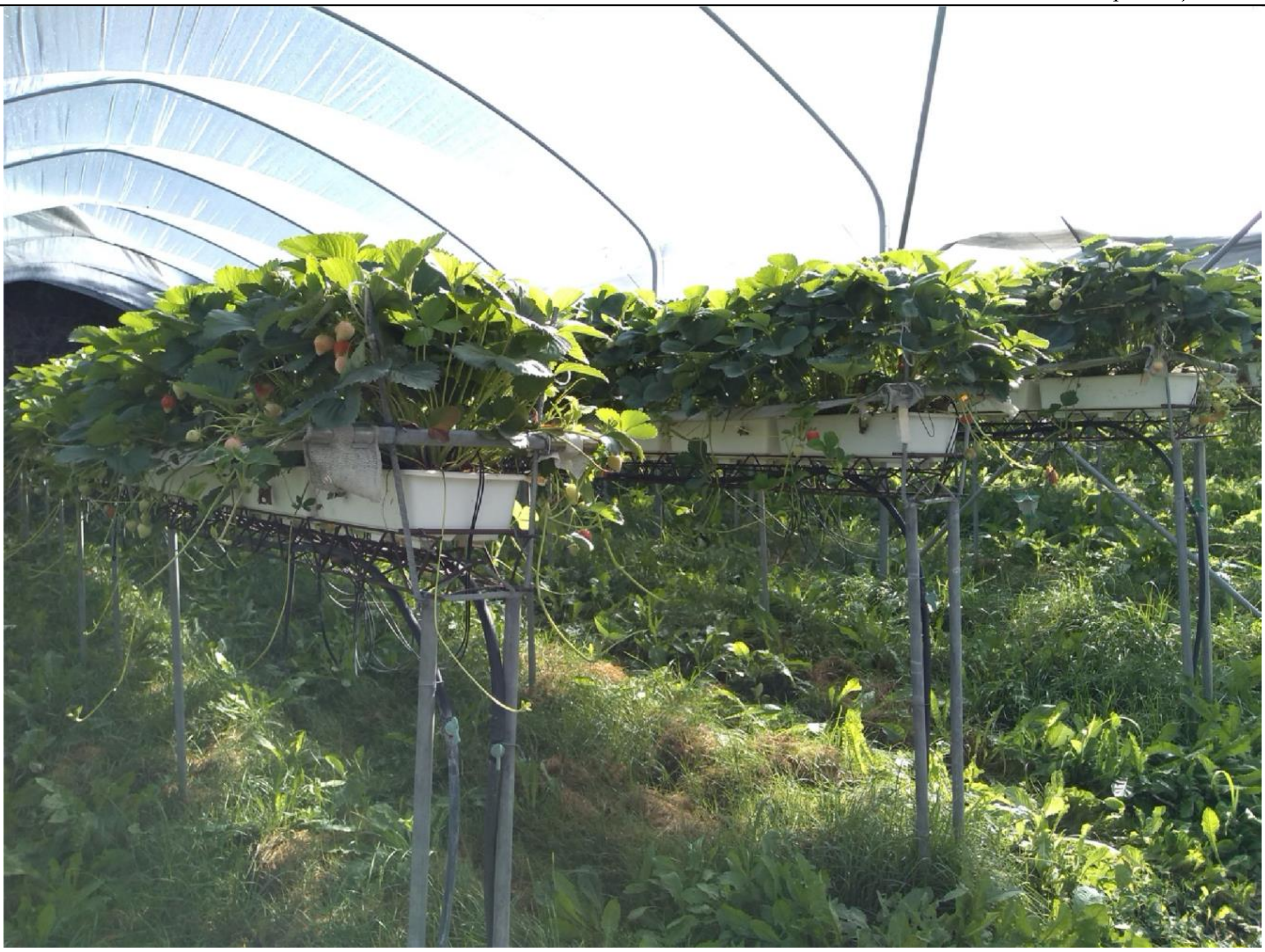

Figure S1. Detail of strawberry field in Drena with strawberry soilless cultivation trays arranged in lines, supported by poles.

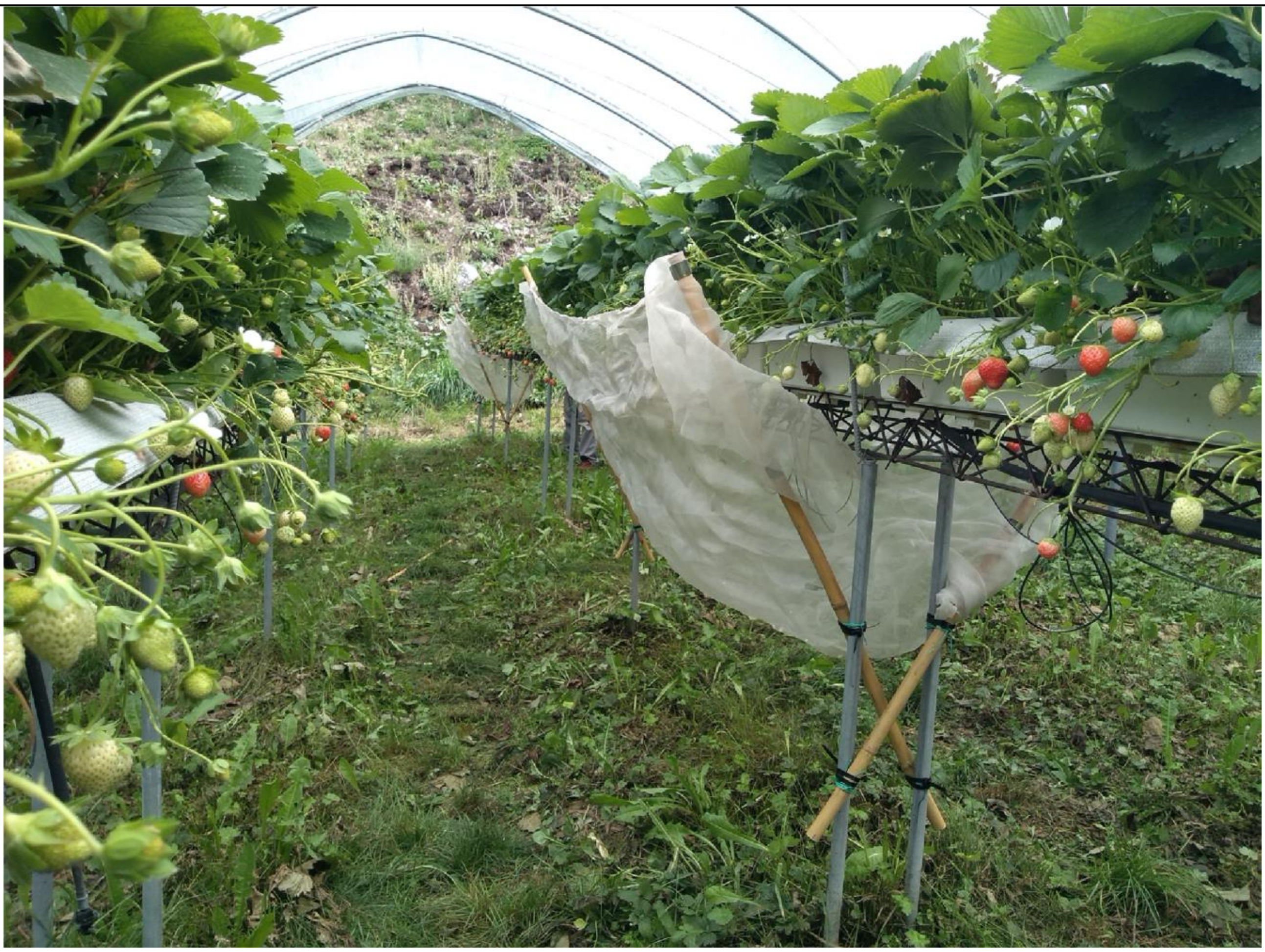

Figure S2. Detail of the hammock set up a with fine net under the tray line structure and above the grass level. They were used in 2019 to intercept and remove the severed flower buds that would have fallen to the ground.

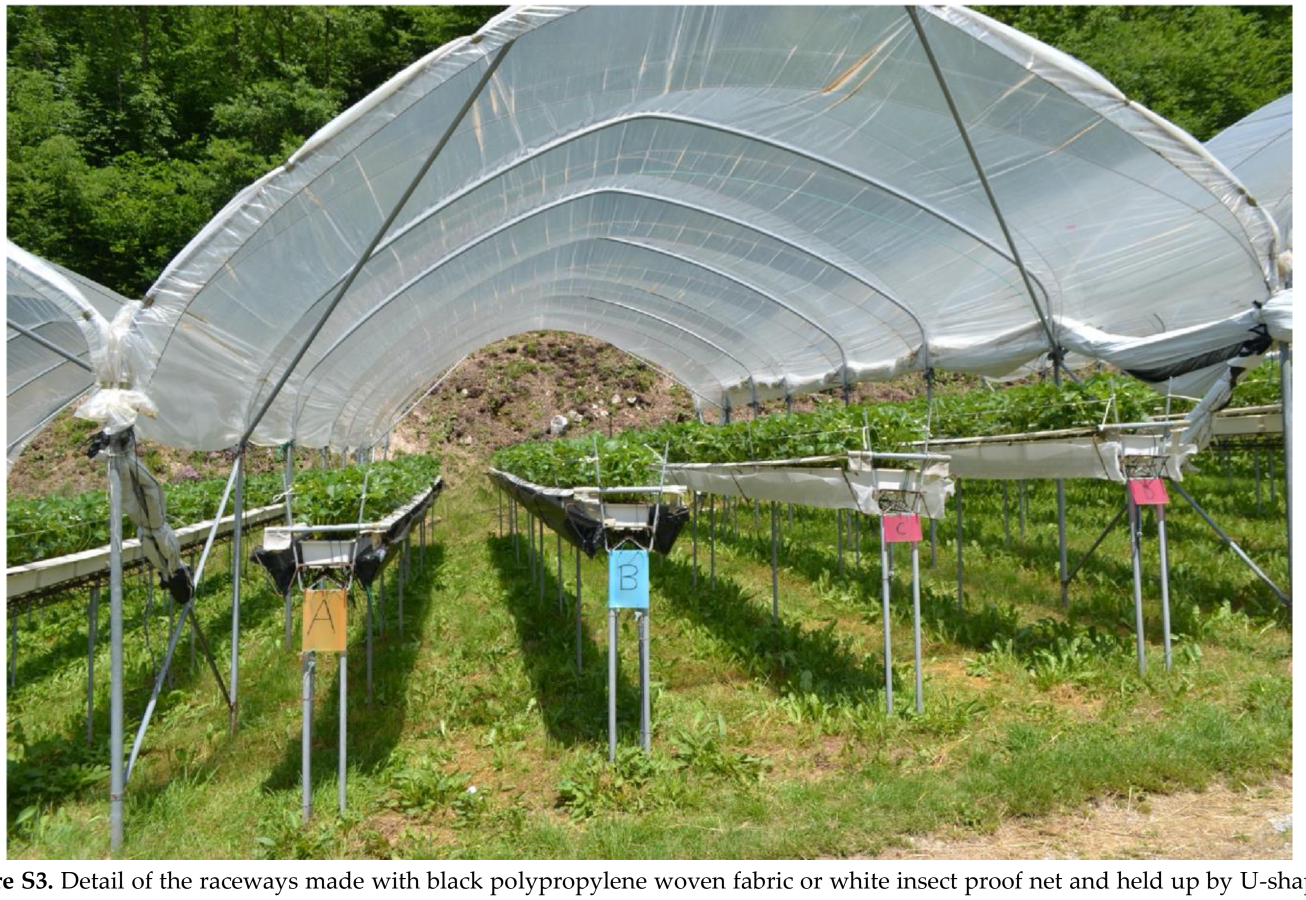

Figure S3. Detail of the raceways made with black polypropylene woven fabric or white insect proof net and held up by U-shaped metallic supports. They were used in 2020 to intercept and remove the severed flower buds that would have fallen to the ground.

Biology of the pest – Results

We found a different developmental time related to the host plants from which they were severed, and for strawberry also to the period of oviposition in the buds ($P<0.01$). The pest development inside the strawberry buds infested in the spring period (May-June) was faster than that observed for adult emergence from the buds attacked between July and September (respectively 28.4±0.9 and 37.7±2.3 days for the 50% of newly emerged adults; Figure S4). Regarding other host plants, 50% of newly emerged adults was reached after 21.2±1.0 days for *R. canina*, 32.8±1.2 days for cultivated blackberry and 69.2±3.7 days for wild blackberry.

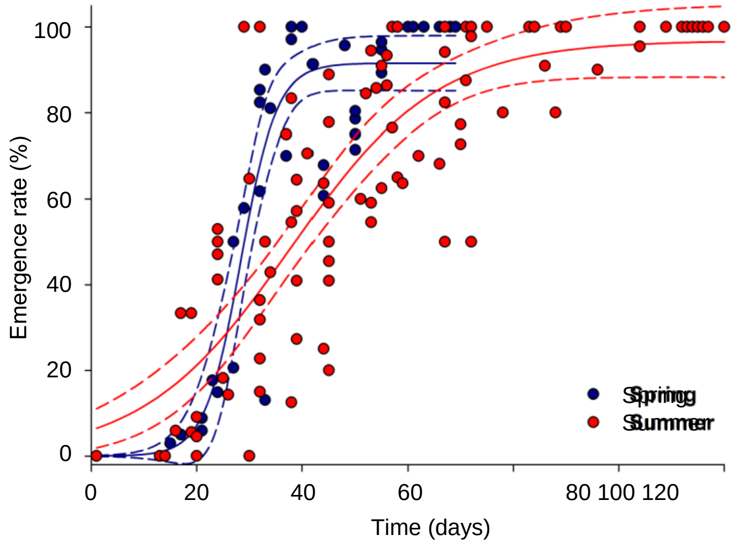

Figure S4. Time-dependent emergence rate for strawberry buds severed in spring (blue) and summer (red) 2019. Dotted lines represent the 95% confidence interval for each three-parameter sigmoid regression curve.

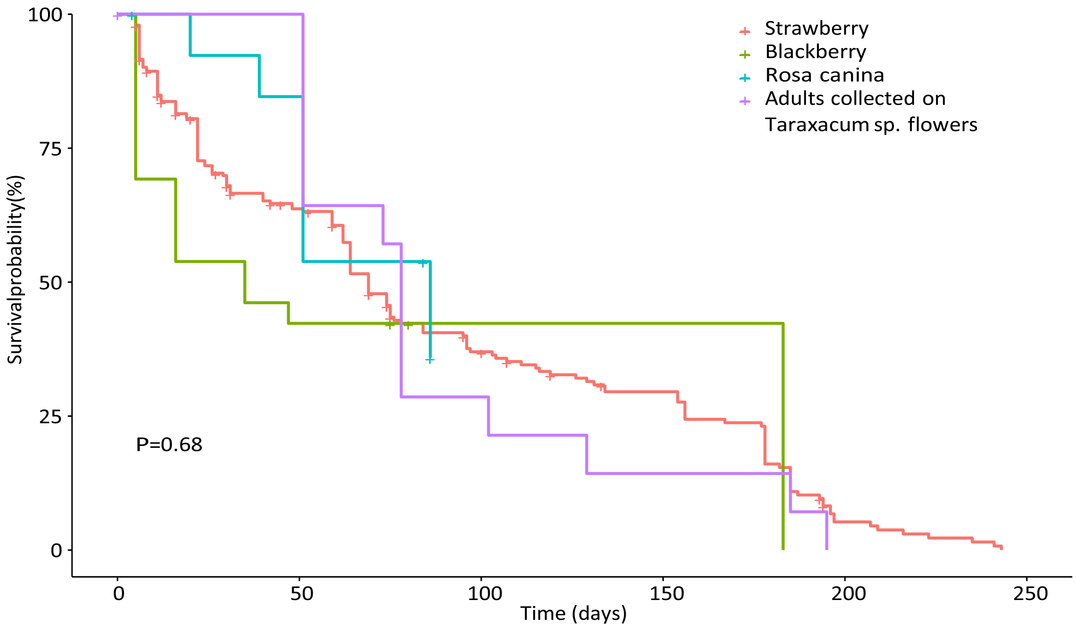

Figure S5. Survival analyses for adults from the 4 different plant species (n= 299 for strawberry, 27 for cultivated blackberry, 14 for *Rosa canina* and 16 for adults collected on *Taraxacum* sp. flowers). Samples from *R. canina* were discarded due to loss of sample integrity (insects had accidentally drowned) after 85 days and data are available until 64% of mortality.

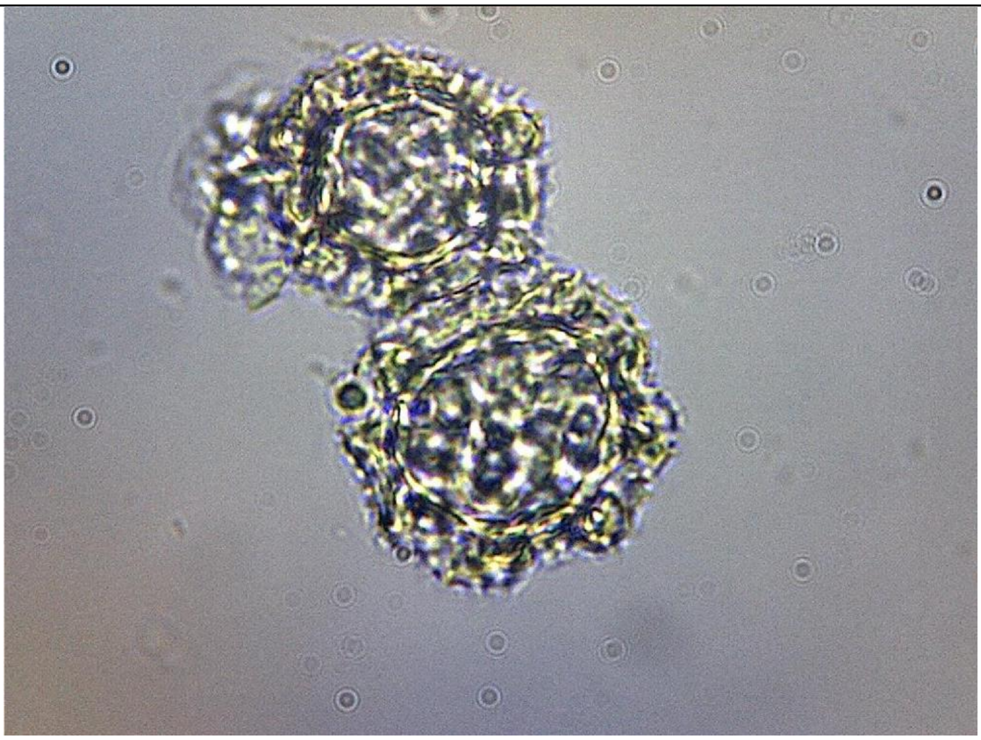

Figure S6. *Taraxacum* sp. pollen grains found within the gut of dissected adults collected on these flowers (microscope photo: Malagnini©).

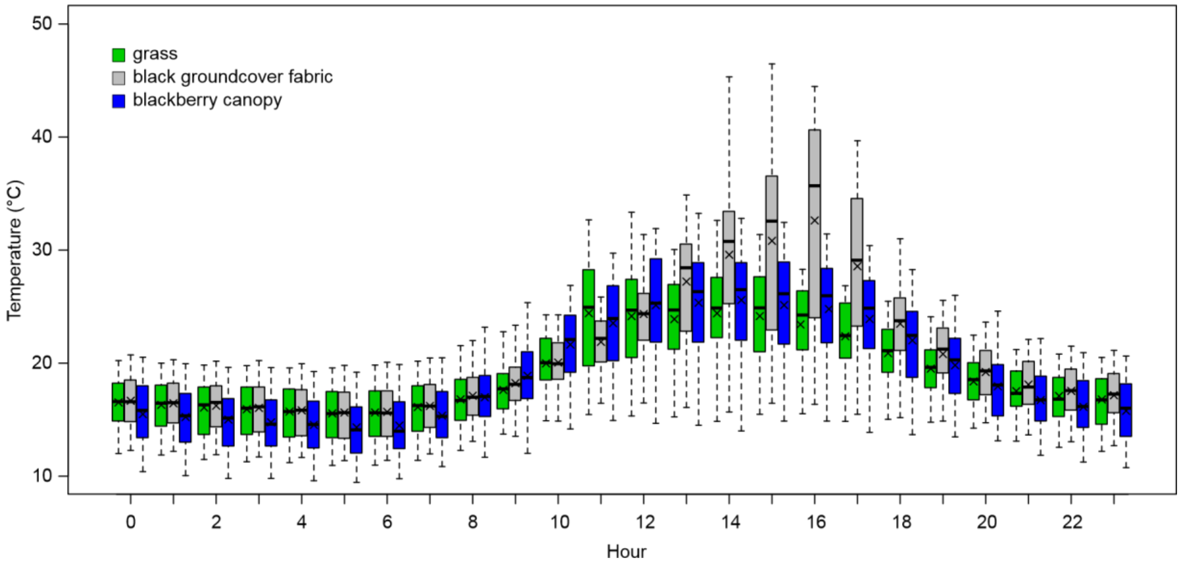

Figure S7. Average temperature recorded on the grass (green), on black groundcover fabric (grey), and within the blackberry canopy (blue), hourly during the period 29th May – 18th July 2020. The boxes enclose the first and third quartiles; the ends of the whiskers represent the 5th/95th percentiles; the solid lines are the median and the cross the mean.

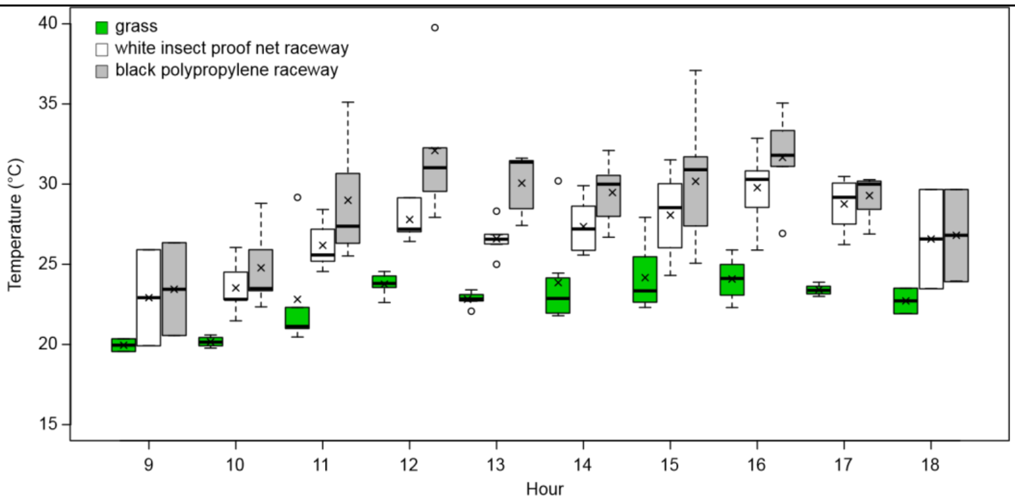

Figure S8. Average temperature recorded on black polypropylene woven fabric raceway (grey), on white insect proof net raceway (white), and on the grass (green), hourly data during the daily hours of summer 2020. The boxes enclose the first and third quartiles; the ends of the whiskers represent the $5_{th}/95_{th}$ percentiles, the solid lines the median and the cross the mean.
